# A Comparison of the Primary Sensory Neurons Used in Olfaction and Vision

**DOI:** 10.3389/fncel.2020.595523

**Published:** 2020-11-05

**Authors:** Colten K. Lankford, Joseph G. Laird, Shivangi M. Inamdar, Sheila A. Baker

**Affiliations:** ^1^Department of Biochemistry, University of Iowa, Iowa City, IA, United States; ^2^Department of Ophthalmology and Visual Sciences, University of Iowa, Iowa City, IA, United States

**Keywords:** olfaction, vision, sensory receptors, photoreceptor, olfactory sensory neuron, GPCR, voltage-gated ion channel, ribbon synapse

## Abstract

Vision, hearing, smell, taste, and touch are the tools used to perceive and navigate the world. They enable us to obtain essential resources such as food and highly desired resources such as mates. Thanks to the investments in biomedical research the molecular unpinning’s of human sensation are rivaled only by our knowledge of sensation in the laboratory mouse. Humans rely heavily on vision whereas mice use smell as their dominant sense. Both modalities have many features in common, starting with signal detection by highly specialized primary sensory neurons—rod and cone photoreceptors (PR) for vision, and olfactory sensory neurons (OSN) for the smell. In this chapter, we provide an overview of how these two types of primary sensory neurons operate while highlighting the similarities and distinctions.

## Introduction

The sensory neurons that initiate olfaction and vision are olfactory sensory neurons (OSN), also referred to as olfactory receptor neurons (ORN), and photoreceptors (PR). Both OSN and PR respond to stimuli using a biochemical signal transduction cascade to trigger changes in membrane potential that alters synaptic transmission. In the absence of an odorant, OSN is basally polarized and when an odorant binds, the OSN depolarize. This results in the generation of an action potential and the release of neurotransmitters. PR have an inverted response. In the absence of light, PR are basally depolarized and hyperpolarize in response to photon absorption. This does not result in an action potential. Instead, graded changes in membrane potential result in graded changes in the amount of neurotransmitter released. Despite the differences in how OSN and PR function there are many similarities in the mechanisms of signaling. Here, we compare these mechanisms as we describe the flow of information from stimulus detection to synaptic transmission in both OSN and PR.

## Anatomy of the Sensory Tissues

### Olfactory Epithelium

**Figure 1:**
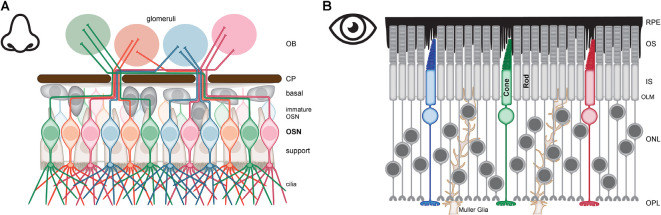
Anatomy of olfactory sensory neurons (OSN) and photoreceptors (PR). **(A)** OSN are defined by the expression of a unique odorant receptor, OSN expressing the same receptor (red, green, blue, or orange) are dispersed throughout the olfactory epithelium (OE) but the axons of these OSN converge to synapse in the same glomeruli of the olfactory bulb (OB). The major compartments of OSN are the cilia for signal detection which extends from an apical dendritic knob, a bipolar cell body for housekeeping functions and housing the genome, and an axon that traverses the cribriform plate (CP) to synapse in the OB. The cell bodies and dendrites of OSN are surrounded by support cells (brown). Basal cells (gray) are stem cells that generate the immature OSN (pale red, green, blue, or orange). **(B)** PR consist of rods for dim light vision (gray) and cones for bright light and color vision (red, green and blue). The major compartments of PRs are organized into four layers—outer segments (OS) for signal detection, inner segments (IS) for housekeeping functions, the nucleus in the outer nuclear layer (ONL) for housing the genome, and the synaptic terminal in the outer plexiform layer (OPL). PR are supported by retinal pigment epithelial (RPE) cells (black) and by Muller Glia (brown).

Primary sensory neurons receive external cues that are relayed to higher cortical centers. These neurons must be exposed to the environment yet protected from damage. OSN are bipolar neurons surrounded by basal and support cells, that together make up the olfactory epithelium (OE) lining the roof of the nasal cavity (Figure 1A; Morrison and Costanzo, 1990). The OE is a component of the peripheral nervous system and is protected by its location deep within the nasal cavity and by a coating of mucus (Whitlock, 2004). The apical, or dendritic, compartment of OSN ends with a dendritic knob from which approximately 5–20 long cilia extend into the mucosal coating to sample inhaled odorants (Menco, 1980b). The basal compartment of OSN narrows to a long, unmyelinated axon that exits the OE to synapse in the olfactory bulb (OB). The OE and bulb are separated from each other by the ethmoid bone. The ethmoid bone has a specialized zone of small openings, the foramina in the cribriform plate (CP), that allow the OSN axons passage to the interior of the skull (Choi and Goldstein, 2018; Norwood et al., 2019). OSNs are genetically encoded to respond to specific odorants based on the single odorant receptor (OR) they express, the axons of OSN expressing the same OR converge on one to two glomeruli and synapse with mitral and tufted cells within the outer nuclear layer (ONL) of the OB (Buck and Axel, 1991; Ressler et al., 1993; Vassar et al., 1993; Mombaerts et al., 1996). The information then flows through the OB directly to the olfactory cortex (Firestein, 2001; Su et al., 2009).

OSN are surrounded by support cells, also known as sustentacular cells. The support cells span the OE with a narrow basal extension and a broad apical surface from which microvilli protrude into the epithelial mucosa (Cuschieri and Bannister, 1975a, b; Firestein, 2001). Mature OSN dendrites appear to be fully enveloped by a single support cell while immature neurons extend dendrites between support cells (Nomura et al., 2004; Liang, 2018). The function of this envelopment remains unknown but the similarities between support cells and the myelinating Schwann cells and oligodendrocytes raises the possibility that these cells function as a “pseudo-myelin sheath” around the OSN dendrite (Liang, 2020). Support cells further assist the OSN with functions typically assigned to glia such as regulation of ion homeostasis and metabolic coupling that provides the glucose needed to power odorant detection in the OSN cilia (Suzuki et al., 1996; Menco et al., 1998; Hegg et al., 2009; Tang et al., 2009; Nunez-Parra et al., 2011; Villar et al., 2017). Despite these glial functions, supporting cells also act as epithelial cells and create a barrier from the external environment. Toxic compounds such as those found in smoke or environmental pollutants are constantly being inhaled. Support cells limit the potential damage to OSN from such compounds in two ways. Diffusion of potentially toxic xenobiotics into the OE is limited by an apical junctional belt formed from the tight junctions and adherens junctions between adjacent support cells (Menco, 1980a; Steinke et al., 2008). Toxic xenobiotics that do get absorbed can be metabolized to reduce harm support cells express higher levels of the cytochrome P450 “detox” enzymes than even the liver (Sarkar, 1992; Getchell et al., 1993; Kern and Pitovski, 1997; Carr et al., 2001).

The third major cell type found in the OE is the basal cells which are stem cells. There are two classes of basal cells: the actively cycling globose basal cells which are the primary regenerative source for the OSN and the quiescent horizontal basal cells which function in renewal for both the OSN and support cells following a substantial injury to the OE (Schwob et al., 2017).

OSN axons leave the OE but still receive support from an adjacent cell. Ensheathing cells surround OSN axon bundles but do not form myelin sheets. Instead, they provide glial support (Ramón-Cueto and Avila, 1998). These cells perform immune functions and help prevent microbial infiltration from the exposed OE from reaching the central nervous system (Leung et al., 2008; Harris et al., 2009). Ensheathing cells also function in OSN renewal, phagocytosing debris from spent OSN, and providing axonal guidance to newly developing OSNs (Doucette, 1990; Su et al., 2013).

### Retina

PR are entirely contained within the retina, the sensory epithelium lining the interior back of the eyeball. Unlike the OE, the retina develops from an outpouching of the forebrain making it a component of the central nervous system (London et al., 2013). The retina is protected by enclosure in the eyeball, and yet, efficiently samples light due to the focus provided by the transparent cornea and lens. The cellular organization of the retina is more complex than the OE. Information flows from PR through two major classes of neurons, bipolar then ganglion cells with lateral signal modulation provided by horizontal and amacrine cells. The ganglion cells are the first action potential firing neuron in this flow of information and their axons bundle together to leave the eyeball as the optic nerve. The optic nerve relays information primarily through the lateral geniculate nucleus of the thalamus to the visual cortex (Masland, 2001; Erskine and Herrera, 2014). There are additional pathways that transmit signals from the retina. These pathways generally route non-image-forming information through the retinohypothalamic tract to communicate to nuclei that control pupil constriction and set the master circadian clock (Foster and Hankins, 2002; Canteras et al., 2011; Szabadi, 2018). Given the complex circuitry of the retina and the amount of signal processing that occurs before information leaves the eyeball it is more accurate to think of the retina (without the PR) as analogous to the OB rather than the OE. Regardless, the similarities in structure, function, and support network for OSN and PR are remarkable.

Like OSN, PR are compartmentalized neurons. Rods are used for vision under dim light and cones are used for vision under bright light as well as providing color vision. Both rods and cones are organized as a linear array of four major compartments: outer segments (OS), inner segments (IS), nucleus, and synaptic terminal (Figure 1B). The OS are the apical-most compartment and are comprised of highly ordered stacks of membranes that are the photosensitive part of the neuron. These membranes house the machinery used to absorb photons and elicit a change in membrane potential. The IS is the cell body and houses organelles required for basic life such as mitochondria, ribosomes, ER, Golgi, endosomes, lysosomes, and proteasomes. This compartment also houses ion transporters and channels integral to setting and maintaining membrane potential. PR nuclei are found in the ONL of the retina (Baker and Kerov, 2013). The nuclei are the widest part of PR and the soma surrounding each nucleus is compressed into thin processes extending both apically and basally for variable distances. This allows for the high packing density of PR and generates the illusion of columns of multiple nuclei when in fact there is just one nucleus per PR with the cell body pushed into the anatomically distinct IS. Rod nuclei, but not cone nuclei, are further distinguished by an inverted arrangement of chromatin such that the dense heterochromatin is concentrated in the center of the nucleus instead of being dispersed in clumps around the periphery. This arrangement is speculated to add to the light-guiding properties of the retina (Solovei et al., 2009; Kreysing et al., 2010). The thin process of soma extending basally from the nucleus serves as the axon but there is no distinct boundary between the end of the IS/soma and the beginning of the axon. The synaptic terminals of rods and cones are housed in the outer plexiform layer (OPL) where they form synapses with horizontal cell processes and bipolar cells dendrites (Lamb, 2013). The rod synaptic terminals are spherical with a single invaginating ribbon synapse while cone synaptic terminals are long and flat, contain tens of invaginating ribbon synapses, and are found in the basal portion of the OPL (Blanks et al., 1974; Okada et al., 1994). The unique structure and function of ribbon vs. conventional synapses will be discussed in a subsequent section.

PR are supported by Muller glial cells and retinal pigment epithelial (RPE) cells. The Muller glia are similar to radial glia. They extend throughout the entire span of the retina and function in multiple ways to support the development, function, and health of the retina. In the region surrounding PR, the Muller glia have numerous short extensions that surround the synapses in the OPL to buffer ion fluxes and clear excess neurotransmitter. The Muller glia also sends extensions into the ONL to provide structural support (Bringmann et al., 2006; Reichenbach and Bringmann, 2013; Wang et al., 2017). Muller glia form adherens junctions with PR demarking the junction between outer nuclear and IS layers (Bunt-Milam et al., 1985; Williams et al., 1990). These junctions and the band of actin filaments running between them form a readily visible line in histological preparations of the retina and historically were given the misnomer of the outer limiting membrane (Williams et al., 1990; Omri et al., 2010). Muller glia extend microvilli past this junctional belt. The proximity of PR and Muller glia throughout the outer nuclear and basal IS layers likely facilitates homeostatic control of ion and nutrient fluxes as well as the glucose-lactate shuttle that fuels the high metabolic demands of PR (Bringmann et al., 2006; Reichenbach and Bringmann, 2013).

Unlike Muller glia, RPE cells are not part of the neural retina but they are intimately associated with PR as they send microvilli from their apical surface into the subretinal space to ensheath PR OS. The RPE provide many essential support functions for PR (Boulton and Dayhaw-Barker, 2001; Strauss, 2005). These include forming the outer blood-retina barrier for protection and filtering nutrients from the vasculature to PR. The RPE also plays a key role in the regeneration of chromophore and renewal of PR OS and these processes will be discussed in more detail in subsequent sections.

## Signaling the Presence of Odorants or Light

Unlike classical neurons that receive inputs from numerous synapses housed in branching dendrites, OSN and PR collect sensory information using modified primary cilia at their apical ends that house the signal transduction machinery for odorant or light detection. Cilia are antenna-like organelles protruding from the cell surface. The simplest type of cilium is a primary cilium which is composed of a microtubule-based axoneme ensheathed by the plasma membrane. They are typically very thin and short (0.2–0.3 μm in diameter and 2–6 μm in length; Satir et al., 2010; Yoon et al., 2019). Individual, non-motile, primary cilia are found on many different cell types, including neurons. Cilia provide a platform for signaling. By protruding from the cell, they allow ready sampling of the local environment and the small diameter of these organelles provides a high surface to the cytoplasmic ratio that allows for a high concentration of receptors and associated signaling molecules. Cilia can house different receptors depending on the cell type and therefore are integral to many cellular signaling pathways in a tissue-type dependent manner. Examples of ciliary-based signaling include pathways regulating development (sonic hedgehog), tissue homeostasis (TGFβ signaling), and neuromodulation (dopamine receptors; Mykytyn and Askwith, 2017; Anvarian et al., 2019).

In the case of sensory perception, the demand for sensitivity is high and this need is met in part by modifications to primary cilia that allow for greater surface area hence more receptors. OSN are modified to grow numerous long cilia; they are not motile but can move with the fluid flow of the nasal mucosa to sample odorants entering the nose. PR are modified such that the ciliary membrane is greatly expanded and folds in and out to form the discs of the OS. In cones, the discs remain continuous with the OS plasma membrane but in rods, the discs are enclosed and separate from the OS plasma membrane. Ciliopathies are multi-syndromic disorders arising from disruptions to the structure or function of primary or modified cilia and consequently, patients often present with loss of smell and blindness among other challenges (McIntyre et al., 2013).

OSN cilia and PR OS each house the biochemical signaling components that convert odorant or photon detection to a change in the resting membrane potential of the neuron. The biochemical signaling cascade in both sensory neurons has many similarities. Both initiate with a G-protein coupled receptor and make use of cyclic nucleotides (cAMP or cGMP) as second messengers to regulate the opening of cyclic-nucleotide gated channels (CNG). However, the response to odorants requires activation of an additional ion channel to generate the electrical response.

### GPCR Signaling in Olfactory Sensory Neurons

GPCRs are characterized by their serpentine structure consisting of an extracellular N-terminus, seven transmembrane domains, and a cytoplasmic C-terminus (Rosenbaum et al., 2009). The subfamily of GPCRs that responds to odorants is collectively known as OR (Buck and Axel, 1991). The odorants that serve as ligands for these receptors are small volatile, mostly hydrophobic, molecules that are inhaled through the nasal passages and diffuse through the olfactory mucosa to reach the OSN cilia. Due to their hydrophobicity, odorants are poorly soluble in the aqueous olfactory mucosa and secreted odorant-binding proteins are thought to serve as carrier molecules to allow odorants to more efficiently reach the OR (Heydel et al., 2013).

ORs make up the largest subfamily of GPCRs; there are 339 functional OR in the human genome and 913 OR in the mouse genome (Godfrey et al., 2004; Malnic et al., 2004). ORs share sequence similarity ranging from 40 to 90% with regions in the third, fourth, and fifth transmembrane domains showing hypervariability (Pilpel and Lancet, 1999; Firestein, 2001). The hypervariable regions are thought to encompass the odorant-binding pocket with the variability providing the molecular basis for the diverse array of odorants that can be recognized by this subfamily. Individual OR are not selective for just one ligand, instead, they can be activated by multiple odorants; a particular OR may be broadly selective and able to be activated by a large number of diverse odorants or they may be more selective with an affinity for a smaller group of odorants with similar chemical signatures (Firestein, 2001; Araneda et al., 2004). While there is no “rose” or “skunk” OR, each OR does have a complex odorant response profile that makes the olfactory system exquisitely sensitive. A study using mixtures of 128 odorants calculated that humans could discriminate at least one trillion distinct odor profiles (Bushdid et al., 2014).

Ligand-activated GPCRs function as guanine nucleotide exchange factors for the cognate heterotrimeric G-protein. Exchange of guanosine diphosphate (GDP) for guanosine triphosphate (GTP) in the alpha subunit of the G-protein causes dissociation of the α subunit from the obligate βγ dimer, either of which may go on to regulate downstream effectors (McCudden et al., 2005). In both the olfactory and visual systems, the α subunit is the primary signal transducer. There are four major subclasses of heterotrimeric G-proteins: G_i/o_, G_s_, G_q_, and G_12_ (Wettschureck and Offermanns, 2005; Melien, 2007). OR signal through G_olf_, a member of the G_s_ or stimulatory subclass of G-proteins (Jones and Reed, 1989).

**Figure 2:**
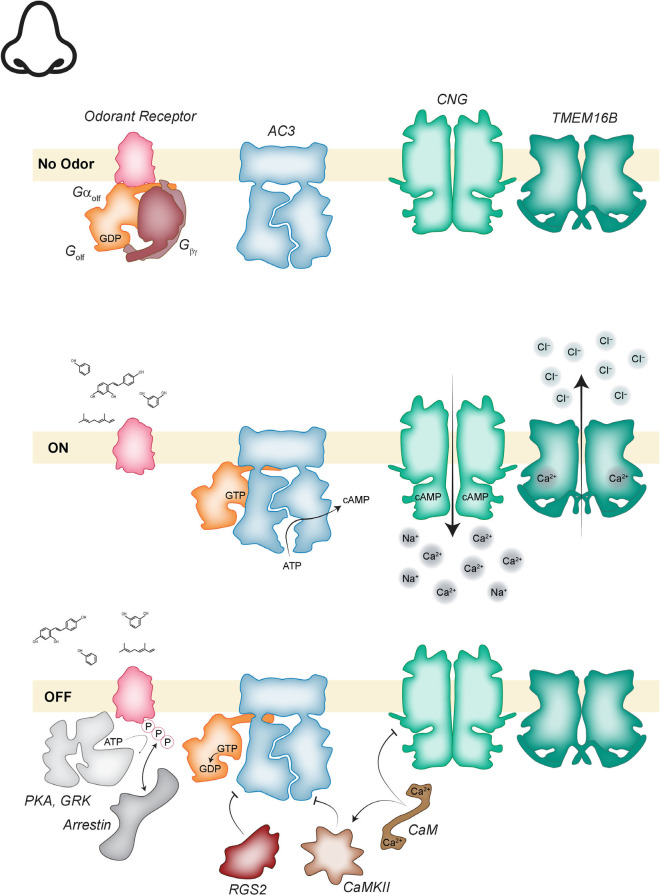
Odorant transduction. (Top) In the absence of odorant, odorant receptors (OR; pink), are associated with the GDP bound heterotrimeric Gα_olf_ (orange, mauve). Adenylate cyclase 3 (AC3; blue) is inactive. cyclic nucleotide gated (CNG; green) and transmembrane protein 16B (TMEM16B; dark green) channels are closed. (Middle) Activation of the cascade begins when odorant binding to an OR activates Gα_olf_ and the GTP bound alpha subunit dissociates to activate AC3. The increase in cyclic adenosine monophosphate (cAMP) concentration opens CNG channels and the resulting influx of calcium opens TMEM16B channels. The cascade is inactivated when Ca^2+^-calmodulin (CaM; brown) directly inhibits CNG channels and indirectly inhibits AC3 *via* calmodulin-dependent kinase II (CamKII; light brown) phosphorylation. AC3 is further inhibited by regulator of G-protein signaling 2 (RGS2; reddish-brown). The OR is phosphorylated by protein kinase A (PKA) and GPCR kinase (GRK; light gray) allowing arrestin (gray) binding which inactivates the receptor.

Following odorant-binding and nucleotide exchange, the GTP-bound Gα_olf_ is free to diffuse laterally along the ciliary membrane to allosterically activate type 3 adenylate cyclase (AC3; Figure 2; Jones and Reed, 1989; Bakalyar and Reed, 1990). Active AC3 generates a high local concentration of cAMP that favors the cAMP-bound, open conformation of the nearby CNG channel which conducts an inward Na^+^-Ca^2+^ current. This inward current is necessary, but only makes a minor contribution to depolarization of the membrane (Kleene, 1993). The added activity of a calcium-activated chloride channel, TMEM16B (also known as anoctamin 2), is required to drive membrane depolarization to the range needed for the generation of an action potential (Kleene, 1993; Stephan et al., 2009). The Ca^2+^ that activates TMEM16B enters the cilia *via* the open CNG channels and the high intracellular concentration of Cl^−^ needed to generate the depolarizing current is maintained by the activity of a Na^+^-K^+^-2Cl^−^ cotransporter (NKCC1; Reisert et al., 2005).

Inactivation of this signaling cascade occurs *via* multiple integrated mechanisms. Consider the activation cascade in reverse, inactivation of TMEM16B will occur when intraciliary Ca^2+^ levels drop. The source of that Ca^2+^ is the open CNG channel. To inactivate the CNG channel, Ca^2+^ mediated feedback is employed. Ca^2+^-bound calmodulin (CaM) can bind to CNG which reduces the channel affinity for cAMP so that even in the presence of cAMP the channel open probability is reduced (Liu et al., 1994). Full inactivation of CNG requires degradation of cAMP, which is accomplished when Ca^2+^-CaM activates the cAMP to AMP conversion activity of phosphodiesterase 1C (Borisy et al., 1992). To keep cAMP levels low AC3 must be inactivated.

AC3 inactivation is the result of the convergence of several signals, inhibitory phosphorylation by CaM-stimulated CaMKII, and an atypical role for an RGS protein (Wei et al., 1998; Sinnarajah et al., 2001). RGS proteins typically function to accelerate the intrinsic GTPase activity of G alpha subunits in the G_i/o_ subfamily. But in the olfactory cilia, RGS2 acts to inhibit the activity of AC3 (Sinnarajah et al., 2001). Finally, Gα_olf_ will hydrolyze GTP, converting it to the inactive GDP bound state which can re-associate with Gβγ.

The final major component of the signal transduction pathway that must be inactivated is the OR. This occurs when the receptor is phosphorylated. There are two kinases involved in this step, G-protein coupled receptor kinase 3 (GRK3) and protein kinase A (PKA; Dawson et al., 1993; Peppel et al., 1997; Mashukova et al., 2006). PKA is activated by the increased intracellular cAMP levels generated by AC3 and GRK3 is recruited by the Gβγ dimer liberated from Gα_olf_ in response to the initial activation of the OR. Phosphorylation of the OR creates a binding site for β-arrestin-2 (Dawson et al., 1993; Boekhoff et al., 1994; Mashukova et al., 2006). This blocks the ability of the OR to activate additional G_olf_ and more dramatically, can trigger internalization of the receptor from the ciliary membrane and translocation to the cell body. To enhance ligand removal, they can be bound by odorant-binding proteins for uptake into the support cells (Strotmann and Breer, 2011). In summary, olfactory signal transduction follows the standard layout of any GPCR signaling pathway with activation occurring *via* a relatively simple linear flow of events and the equally important inactivation steps occurring *via* multiple mechanisms that include a role for Ca^2+^ feedback at each step.

### GPCR Signaling in Photoreceptors

OSN are defined by the specific OR expressed in the cilia and a similar definition can be made for PR by considering the GPCR expressed in the OS. Rods use rhodopsin, the apo form is referred to as opsin while “rhodopsin” specifically refers to the halo form with the ligand-binding pocket occupied by the obligate co-factor, 11-cis retinal, a derivative of vitamin A (Saari, 2016). Rhodopsin is most sensitive to green light with an absorption maximum at 500 nm (Hubbard, 1969). Despite this sensitivity to green light, information from rods is not used to compute color information. Color vision is derived from information received from cone PR, and the number of cone types expressing spectrally tuned opsins defines the range of hues that can be perceived. Most mammals are dichromats, meaning the retina of these animals has two types of cones. These are usually a UV-blue sensitive cone expressing a short-wavelength opsin (OPN1SW) tuned to the light of 410–435 nm (Hunt et al., 2001), and a green-red sensitive cone expressing a medium to long-wavelength opsin (OPN1MW or OPN1LW) tuned to the light of 500–570 nm (Mollon, 1999; Yokoyama et al., 2008; Shichida and Matsuyama, 2009). Dichromats can distinguish many different hues of blue-gray-yellow (Roth et al., 2007; Pridmore, 2014). Trichromats, which for mammals are limited to humans and a few other old-world primate species, have three cone types. These include a blue-sensitive cone expressing OPN1SW, a green-sensitive cone expressing OPN1MW, and a red-sensitive cone expressing OPN1LW (Nathans et al., 1986; Mollon, 1999; Solomon and Lennie, 2007). The addition of this one cone greatly expands the range of color vision such that humans can distinguish about 2.3 million hues (Linhares et al., 2008). Red-green color blindness is a common disorder inherited in an x-linked recessive pattern that affects about 8% of Northern European-descended men (Deeb, 2005). It is more appropriate to call such individuals color-deficient since they do not lose all color vision. Instead, they see the world in the same way as other dichromatic mammals.

**Figure 3:**
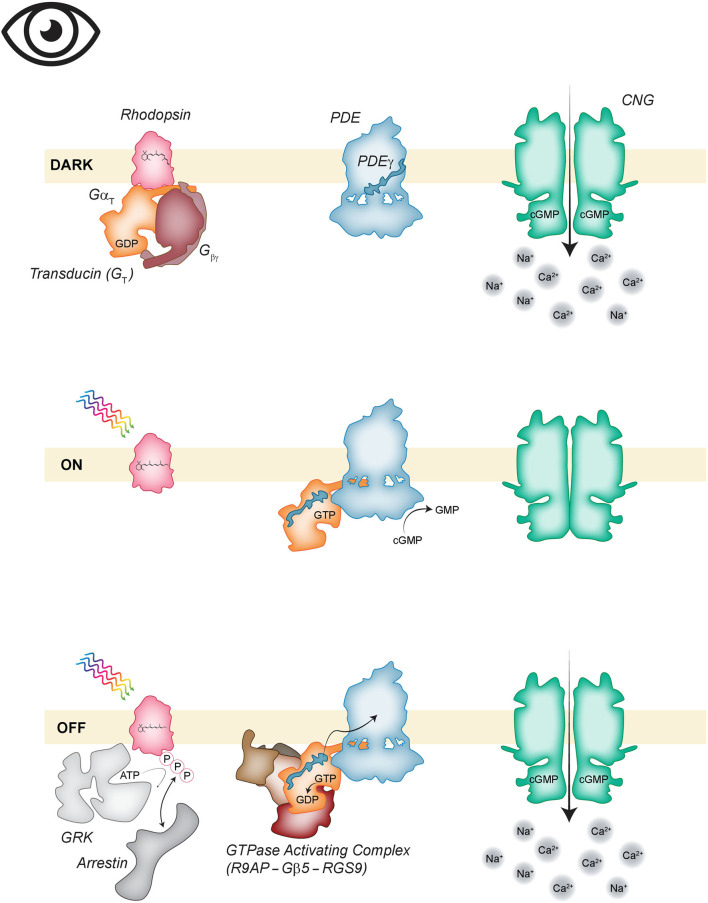
Phototransduction. (Top) In the absence of light, inactive, 11-cis retinal bound, rhodopsin (pink) is associated with the GDP bound heterotrimeric Gα_T_ (orange, mauve). Phosphodiesterase 6 (PDE6; blue) is basally autoinhibited. Guanylate cyclase (not shown) generates cyclic guanosine monophosphate (cGMP) that keeps CNG channels (green) open. (Middle) Activation of the cascade begins when photon absorption isomerizes 11-cis-retinal to the all-trans conformation. The activated rhodopsin activates Gα_T_; the GTP bound alpha subunit dissociates to bind PDEγ thus removing the autoinhibition of PDE6. The decrease in cGMP concentration closes CNG channels. (Bottom) The cascade is inactivated when the GTPase Activating Complex consisting of R9AP (brown), Gβ5 (light brown), and RGS9 (reddish-brown) activates GTP hydrolysis on Gα_T_. Rhodopsin is phosphorylated by GRK (light gray) allowing arrestin (gray) binding which inactivates the receptor.

The phototransduction cascade from activation of GPCR to altered membrane potential is conceptually similar to the odorant-transduction pathway in OSN but distinct with the major changes being the use of an inhibitory instead of a stimulatory class of G-protein, a different effector enzyme, the lack of a calcium-activated chloride channel, and the ultimate effect of closing rather than opening a CNG channel (Figure 3). For the sake of simplicity, we will not continue to distinguish between the different isoforms of the proteins involved in rod vs. cone phototransduction. When PR are in the dark-adapted resting state, cGMP levels are high due to the activity of a membrane-bound guanylate cyclase which generates cGMP and the low basal activity of the countering enzyme phosphodiesterase 6 (PDE6) that degrades cGMP to GMP. PDE activity is low due to autoinhibition by the gamma subunit of the enzyme (PDEγ). cGMP is an activator of the nearby CNG channels found at the edge of cone discs or in the plasma membrane surrounding rod discs. The inward Na^+^/Ca^2+^ current or “dark current” carried by CNG is what keeps PR depolarized at rest (Arshavsky et al., 2002; Arshavsky and Burns, 2012; Michalakis et al., 2018). This is opposite to the resting state in OSN where CNG channels are closed.

Activation of the phototransduction cascade occurs when photon absorption causes isomerization of the rhodopsin-bound chromophore, from 11-cis retinal to all-trans-retinal. This forces rhodopsin into the activated conformation. Activated rhodopsin transduces the signal by exchanging GDP for GTP in the alpha subunit of transducin or G_T_ which belongs to the G_i/o_ subclass of heterotrimeric G-proteins. The GTP-bound Gα_T_ dissociates from Gβγ, diffuses along the disc membrane, and binds the gamma subunit of the effector enzyme, PDE6. Association with Gα_T_ relieves inhibition of PDE which can then catalyze the degradation of cGMP to GMP (Arshavsky and Burns, 2014). Decreased cGMP concentrations lead to the closure of CNG channels, inactivating the dark current and driving the cell to hyperpolarize (Arshavsky et al., 2002; Arshavsky and Burns, 2012).

Inactivation of the phototransduction cascade requires inactivating each component like what occurs in OSN. To reopen the CNG channels cGMP levels must increase thus PDE activity must be inhibited. This is accomplished by inactivating transducin so that PDEγ can be released. Inactivating Gα_T_ is the rate-limiting step of inactivation and is accomplished by a GTPase accelerating complex composed of RGS9, Gβ5, and R9AP (Krispel et al., 2006; Arshavsky and Wensel, 2013). RGS9 enhances the GTPase activity of Gα_T_ and the Gβ5-R9AP subunits serve to regulate the stability, targeting, and membrane localization of RGS9 (Arshavsky and Wensel, 2013). Once GTP is hydrolyzed to GDP, Gα_T_ can re-associate with Gβγ.

Ultimately the GPCR must be inactivated and that process for rhodopsin is similar to that for the ORs. Rhodopsin is targeted by G-protein coupled receptor kinase (GRK1, also called rhodopsin kinase) which phosphorylates the cytoplasmic C-terminal tail of rhodopsin to create a binding site for arrestin. This blocks the ability of rhodopsin to activate additional G_T_ but it does not trigger internalization as is seen with ORs—rhodopsin remains in the disc membranes of the OS (Burns and Arshavsky, 2005; Arshavsky and Burns, 2012).

A major difference between the inactivation of OR and rhodopsin is that odorants reversibly bind to OR and release as concentrations drop while the chromophore for rhodopsin must be regenerated into the 11-cis isomer to absorb another photon. The regeneration of chromophore is a multi-enzymatic pathway called the visual cycle. Some steps of the visual cycle occur in PR OS but the key step of isomerizing the bond at the 11th carbon of retinal from the trans to cis confirmation is carried out by an isomerase found in the adjacent RPE cells. Interestingly, there is a secondary visual cycle thought to primarily serve cones where the key isomerase is found in the other major support cell for PR, the Muller Glia (Tsin et al., 2018).

As with odorant signaling, calcium feedback is also involved in inactivating the phototransduction cascade. Ca^2+^ influx through CNG channels is countered by Ca^2+^ extrusion through the coupled K^+^-Na^+^/Ca^2+^ ion exchanger (NCKX) which continues after CNG channels close. This results in a light-induced drop in intracellular Ca^2+^ concentration. Intracellular Ca^2+^ levels regulate phototransduction at two steps through two different Ca^2+^ binding proteins: recoverin and guanylate cyclase-activating protein (GCAP). Recoverin in the Ca^2+^ bound state inhibits GRK1, but when the calcium levels drop this inhibition is released and GRK1 phosphorylates rhodopsin to inhibit further signaling. GCAP is also inhibited when bound to Ca^2+^, but as active phototransduction results in decreased Ca^2+^ levels, free GCAP activates guanylate cyclase (GC; Burns and Arshavsky, 2005; Arshavsky and Burns, 2012). GC produces cGMP which can bind to CNG channels and reestablish the dark current.

### Sensory Adaptation

The ability of the olfactory and visual systems to undergo sensory adaption—the reduction of sensory sensitivity with continued stimulation—is striking. We experience this adaption daily as we go “nose-blind” to powerful odors such as our perfume or air fresheners and have to wait for our “eyes to adjust” to sudden changes in brightness such as encountered when walking out of a dim lecture hall into a brightly lit lobby. Sensory adaptation is a complex process involving changes at multiple levels of circuitry and the level of stimulus detection by the primary sensory neurons (Demb, 2008; Pellegrino et al., 2017). The chief mechanism of adaptation in both OSN and PR is feedback inhibition of the ciliary signaling cascades.

Feedback inhibition of the odorant transduction cascade employs the tools of inactivation so that the response becomes reduced with prolonged stimulation. In response to odorants, calcium entering the cilia through open CNG channels is bound by calmodulin which is a central coordinator of feedback inhibition. Ca^2+^-calmodulin activates phosphodiesterase 1C which degrades cAMP while signaling through CaMKII to inhibit AC3 thus preventing synthesis of new cAMP molecules (Borisy et al., 1992; Yan et al., 1995; Wei et al., 1998; Cygnar and Zhao, 2009). In addition to modulating the intra-ciliary levels of cAMP, Ca^2+^-calmodulin directly desensitizes CNG channels so that *more* cAMP is required to open the channels and it is thought that this is the primary mechanism of adaptation (Chen and Yau, 1994; Kurahashi and Menini, 1997). A second, dramatic way to reduce the response to any given combination of odorants is to remove the receptors from the ciliary membrane. This is accomplished when phosphorylation of the OR creates a binding site for β-arrestin-2. This triggers clathrin-mediated internalization of the receptor from the ciliary membrane into endosomes residing in the cell body (Mashukova et al., 2006). By reducing the available receptor as well as dampening its ability to transduce an effect, the signal generated by continued odorant exposure is reduced.

The ability to desensitize the response to odorant has two very practical outcomes. It allows other OSN to respond to a changing odorant landscape as newly introduced odors will elicit relatively stronger signals. This means that instead of being limited to responding to one strong odorant, a complex odorant profile can be detected which provides a deeper, more accurate perception of the environment. Second, it is essential to desensitize the response to very noxious odorants. Afterall, a common response to walking into a space that smells horrible is to hold our breath. That is not a sustainable solution.

Adaptation to prolonged light is fundamentally different from adaptation to prolonged odorants even though similar mechanisms are employed. Adaptation involves reducing PR sensitivity to prevent saturation and increases temporal resolution. Rods are so sensitive they can report the presence of a signal photon and become saturated in medium light intensity environments. Cones are less sensitive and can adapt so that they essentially do not saturate. The combination of these two systems creates a dynamic range covering ~11 orders of magnitude (Pugh et al., 1999; Govardovskii et al., 2000; Arshavsky and Burns, 2012).

It can be tempting to assume light adaptation in rods is not needed since the cone system takes over in bright light. However, rods can respond to a range of ~1–10,000 photons per second, but without adaptation, it has been estimated that the rods would become unresponsive at just ~100 photons per second (Govardovskii et al., 2000). Light adaptation consists of cellular desensitization, acceleration of response inactivation, and extension of the operating range. All three of these phenomena are the result of multiple converging mechanisms that include calcium-dependent and independent processes (Pugh et al., 1999; Govardovskii et al., 2000; Arshavsky and Burns, 2012). For the sake of comparison to OSN where Ca^2+^-calmodulin plays a major role, we will just highlight the major calcium-dependent processes in PR.

Light causes a transient drop in intracellular calcium since entry *via* CNG channels is decreased. There are three major calcium-sensing proteins active in PR, GCAPs, Ca^2+^-calmodulin, and recoverin. GCAP in the calcium-free state activates GC and increases levels of cGMP, this is the major route by which the operating range of PR is increased (Mendez et al., 2001; Burns et al., 2002). Range extension is also influenced by the release of CNG inhibition by Ca^2+^-calmodulin (Weitz et al., 1998). The effect of Ca^2+^-calmodulin on CNG channels in OSN and PR is the opposite because of the fundamental difference in the open state of the CNG channels at rest in these two sensory neurons. Both GCAPs and regulation of CNG are at play in cones, however, cone CNG channels are regulated by CNG-modulin instead of Ca^2+^-calmodulin (Rebrik et al., 2012). Recoverin can function indirectly by buffering calcium and directly by inhibiting GRK—the release of this inhibition in low calcium will result in GRK binding to and phosphorylating active rhodopsin, thus inactivating the receptor and causing desensitization (Pugh et al., 1999; Higgins et al., 2006; Komolov et al., 2009; Chen et al., 2012).

Finally, where OSN can be desensitized by translocation of the ORs, this does not happen for either rhodopsin or cone opsins. However, there are three proteins in PR that do undergo translocation—transducin, recoverin, and arrestin (Arshavsky, 2003; Calvert et al., 2006). In response to light, transducin moves from the OS to the IS (Sokolov et al., 2002; Majumder et al., 2013). This reduces activation of PDE and thereby degradation of cGMP so results in reducing sensitivity to prolonged light exposure. Recoverin also moves out of the OS in response to light. This reduces the inhibition of GRK, allowing more efficient phosphorylation of activated rhodopsin which enhances arrestin recruitment (Strissel et al., 2005). Arrestin moves in the opposite direction. In response to light, it moves into the outer segment (Broekhuyse et al., 1985; Mirshahi et al., 1994). Increased arrestin in proximity to activated rhodopsin will blunt the response to prolonged light exposure and accelerate recovery.

## Propagating the Electrical Signal

OSN and PR are bipolar neurons and the electrical signal initiated in the apical cilia or outer segment must reach the basal synapse to alter neurotransmitter release and communicate the signal onto the next neurons in the circuit. OSN and PR achieve this by two different means with both requiring the coordinated action of voltage-gated ion channels. OSN are firing neurons and the depolarization initiated by the odorant signaling cascade triggers a depolarizing wave, or action potential, that propagates down the axon. This action potential is an all or nothing event, similar to flipping a light switch. Action potentials are of consistent strength and duration, but the frequency of firing is proportional to the amount of stimulation, i.e., odorant concentration (Rospars et al., 2003). Conversely, PR do not fire action potentials. Instead, the synaptic output is responsive to graded changes in membrane potential (Rodieck, 1998). The overall PR response is more like dialing up or down a dimmer switch where stimulus intensity or duration is proportional to the activation state of the phototransduction cascade.

### OSN Voltage Response

Under basal conditions, that is in the absence of odorant, ORs are polarized. An exact measure of the resting membrane potential of OSN has proven difficult to obtain, but there seems to be a consensus that it lies between −75 and −50 mV (Dubin and Dionne, 1994; Narusuye et al., 2003; Pun and Kleene, 2004). Activation of the odorant-transduction cascade depolarizes the OSN which is translated into an action potential that propagates down the axon to the synapse. While the exact complement of ionic currents found in vertebrate OSN varies slightly across species, the general mechanism of action potential firing is conserved.

Depolarization triggers the opening of voltage-gated sodium (Nav) channels along the length of the axon (Narusuye et al., 2003). In the majority of species examined, these sodium channels are tetrodotoxin sensitive and studies in rodents revealed the principle channel to be Nav1.7 (Narusuye et al., 2003; Ahn et al., 2011). Nav1.7 is critical for OSN function and Nav1.7 knockout mice exhibit anosmia due to failure of the OSN to generate a synaptic signal despite still generating action potentials. This suggests that Nav1.7 is not required for action potential generation but instead is involved in the propagation of the action potential to the synaptic terminal. Other Nav channels including Nav1.3 and Nav1.5 are expressed in OSN and Nav1.3 localizes to the axon suggesting a potential function in action potential generation (Weiss et al., 2011; Bolz et al., 2017). Regardless of the molecular identity of the Nav channels involved, the inward Na^+^ flux further depolarizes the membrane driving the rising phase of the action potential. In response, voltage-gated potassium (Kv) channels, of the delayed rectifier and A-type, open, and the resulting K^+^ efflux repolarizes the membrane of the axon (Narusuye et al., 2003). In some species, Ca^2+^ currents also contribute to the action potential. In the newt *Cynops pyrrhogaster* OSN, T-type Cav channels open at more negative potentials than the axonal Nav channels and the resulting Ca^2+^ current enhances OSN sensitivity by reducing the threshold required to generate an action potential (Kawai et al., 1996).

The ion channels responsible for setting resting membrane potential are poorly defined (Frenz et al., 2014). But OSN are intrinsically noisy due to the presence of basal electrical activity even in the absence of a stimulating odorant (Lowe and Gold, 1991; Reisert, 2010). This basal electrical activity shapes the resting membrane potential and regulates the firing capacity of the OSN (Reisert, 2010). Spontaneous firing occurs at a rate up to 3 Hz compared to the ~30–50 Hz maximal rate evoked by odorant binding to OR (Reisert and Matthews, 1999; Rospars et al., 2003). A component of this basal activity is due to the spontaneous opening of Nav1.5 channels in the dendritic knob (Dionne, 2016). The basal activity is thought to prime the response to odorant-binding in mature OSN and facilitate axon development in immature OSN as well as stabilize the connection with postsynaptic mitral and tufted cells (Yu et al., 2004; Nakashima et al., 2013; Dionne, 2016).

While the major mechanism of OSN adaptation occurs at the ciliary signaling cascade as described, OSN also experiences spike frequency accommodation where the action potential frequency generated by a depolarizing current is reduced with time. This is thought to be due to the action of a Ca^2+^ gated K^+^ channel as pharmacological inhibition of this channel impedes OSN accommodation (Kawai, 2002).

### PR Voltage Response

Unlike OSN, PR are not firing neurons and the graded voltage response generated by phototransduction directly controls the synaptic output. However, this does not occur without modulation and the PR voltage response is shaped by voltage-gated ion channels in the inner segment, primarily, heteromeric Kv2.1/Kv8.2 channels and HCN1 channels (Bader et al., 1982; Barnes and Hille, 1989; MacLeish and Nurse, 2007).

Kv2.1 is a shab-like Kv channel broadly expressed in the nervous system while Kv8.2 is a regulatory subunit expressed in photoreceptors (Pinto and Klumpp, 1998; Bocksteins, 2016; Gayet-Primo et al., 2018). Assembly of Kv2.1 with Kv8.2 creates a channel that activates at more negative potentials and is slower to inactivate compared to Kv2.1 alone (Barnes, 1994; Czirják et al., 2007; Gayet-Primo et al., 2018). The current carried by this hybrid channel is referred to as *I*_kx_. *I*_kx_ is the primary hyperpolarizing current at rest. In combination with the electrogenic activity of Na^+^/K^+^-ATPase in the IS and NCKX in the OS, *I*_kx_ directly opposes the depolarizing dark current carried by the CNG channels in the OS (Beech and Barnes, 1989; Barnes, 1994; Moriondo and Rispoli, 2010; Hart et al., 2019). In the larger PR of amphibians, but not in rodent PR, an additional outward K^+^ current carried by Ca^2+^ activated BK channels (*I*_KCa_) is thought to help clamp the resting membrane potential (Moriondo et al., 2001; Xu and Slaughter, 2005; Pelucchi et al., 2008; Tanimoto et al., 2012). Altogether, the activity of these channels and transporters results in a resting (dark) membrane potential of about −35 mV (Beech and Barnes, 1989).

Recovery from light-induced hyperpolarization is mediated by both Kv2.1/Kv8.2 and HCN1 channels which operate over different voltage ranges and thus lighting conditions. *I*_kx_ has a half activation voltage of −46 mV and Kv2.1/Kv8.2 channels open in the dark begin closing as the PR hyperpolarizes. Kv2.1/Kv8.2 channels are most sensitive to voltage changes between −35 and −50 mV where even small voltage changes will have a significant impact on the number of channels open. Thus, *I*_kx_ inactivation occurs even in response to the dim light that weakly hyperpolarizes the PR membrane. Reduction of the hyperpolarizing *I*_kx_ facilitates PR recovery to the depolarized, dark state (Beech and Barnes, 1989; Gayet-Primo et al., 2018). Under bright sustained light, the PR membrane potential reaches further hyperpolarization which triggers the opening of the hyperpolarization gated HCN1 channels which have a half activation voltage of −75 mV. HCN1 channels carry an inward mixed cation current referred to as *I*_h_. This current is the primary driver of depolarization and functions to quickly push the PR back toward the resting depolarized state (Bader et al., 1979; Baylor et al., 1984; Beech and Barnes, 1989; Barrow and Wu, 2009). Full recovery back to the dark state is achieved when phototransduction inactivates and CNG channels open to reestablishing the dark current. Reactivation of the dark current is slower than *I*_h_ activation, and while the dark current alone is sufficient to return the PR to the dark state, recovery is delayed without *I*_h_. Prolonged PR hyperpolarization in the absence of *I*_h_ results in saturation of the downstream neural circuits, preventing them from properly modulating and carrying on the signal (Knop et al., 2008; Seeliger et al., 2011).

In summary, Kv2.1/Kv8.2 channels play a prominent role in setting the dark-adapted resting membrane potential. In response to light-driven hyperpolarization, Kv2.1/Kv8.2 works in concert with HCN1 to exert temporal control over rod output. While the voltage modulation in the PR differs from the action potential firing in OSN, they are similar in that both involve coordination of opposing currents carried by voltage-gated ion channels to drive the activation and recovery of the voltage response.

### Conventional vs. Ribbon Synapses

**Figure 4:**
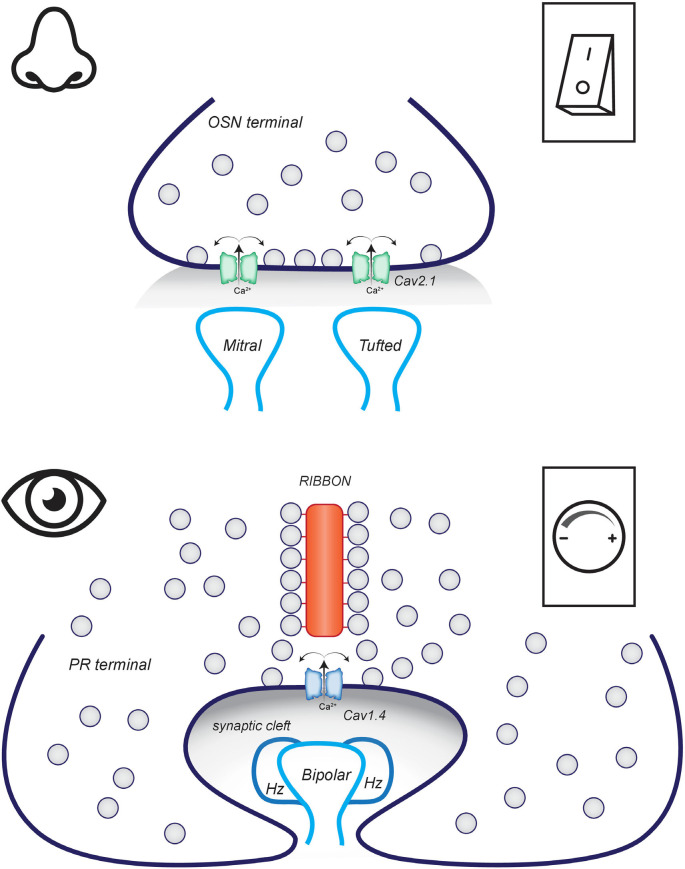
Conventional vs. Ribbon synapses. (Top) OSN make conventional flat synapses with the dendrites of mitral and tufted cells in the OB. The arrival of an action potential depolarizes the membrane. This triggers the opening of Cav2.1 channels and the resulting calcium influx triggers the fusion of synaptic vesicles. (Bottom) PR terminals are invaginated with bipolar dendrites and horizontal cell processes forming a triad synapse. In the dark, Cav1.4 channels are open and synaptic vesicles are fusing to release neurotransmitters. Activation of the phototransduction cascade causes graded hyperpolarization of the membrane which causes Cav1.4 channels to close and synaptic vesicle fusion to slow.

When an action potential reaches the presynaptic terminal of the OSN in the OB, N-type Cav2.2 channels open and the resulting influx of Ca^2+^ triggers the fusion of synaptic vesicles with the plasma membrane and release of glutamate into the synaptic cleft (Figure 4; Weiss et al., 2014). AMPA and NMDA receptors on the post-synaptic dendrites of mitral and tufted cells are activated and the signal propagates through the OB. It should be noted that OSN are not homogenous and one subpopulation can be identified by expression of an alternate synaptic Cav channel, the P/Q-type Cav2.1 channel (Pyrski et al., 2018). Unraveling the function of this OSN subpopulation will be required to understand the physiological significance of expressing Cav2.2 vs. Cav2.1 in the synapse. Presynaptic terminals require more than just a correctly tuned Cav channel and synaptic vesicles to communicate. The molecular architecture of OSN terminals is to our understanding poorly defined. But we can make inferences based on the organization of other conventional synapses.

Conventional presynaptic terminals are host to a collection of proteins known as the cytomatrix at the active zone (CAZ); this includes bassoon, piccolo, CAST, ELKS, RIM, RIM-binding proteins, Munc13, and liprins (Szule et al., 2015). CAZ components share overlapping roles in providing structural organization to the active zone and in regulating several aspects of the synaptic vesicle cycle including tethering, priming, and fusion. This network of proteins can be dynamically regulated by phosphorylation, altered gene expression, or enhanced protein degradation so that individual synapses can tune firing rates in response to overall neuronal network activity (Lazarevic et al., 2013; Torres and Inestrosa, 2018). Individual synapses may be further distinguished by the exact complement of CAZ proteins expressed in that terminal since many of the CAZ proteins exist as small multi-gene families subject to cell-type-specific expression and alternative splicing. Further investigation is needed to determine if any subpopulations of OSN presynaptic terminals may be molecularly and functionally distinguished based on the potential diversity provided by the CAZ complex.

The PR synapse is not like the conventional synapse of OSN (Figure 4). It is an invaginating ribbon-type synapse. Ribbon synapses are defined by the presence of a ribbon organelle that tethers synaptic vesicles adjacent to the active zone. This type of synapse is rare but not unique to PR. Ribbon synapses are also found in the bipolar neurons downstream of PR and in auditory and vestibular hair cells. These synapses are related in that they release neurotransmitters in direct correlation to graded changes in membrane potential instead of in response to an action potential (Matthews and Fuchs, 2010). Like any other synapse, the active zone of a ribbon synapse is marked by clusters of voltage-gated calcium channels that generate the high calcium microdomains that trigger the fusion of synaptic vesicles with the plasma membrane. In PR ribbon synapses, this calcium channel is the L-type Cav1.4 (Bech-Hansen et al., 1998; Mansergh et al., 2005). Two major features distinguish Cav1.4 from the Cav2.2 channels in OSN and make them better matched to the physiology of PR. First, Cav1.4 opens at more negative potentials, so conducts current in the dark-adapted PR and the channel is responsive to small changes in membrane potential in the range elicited by smaller changes in light intensity or duration. Second, Cav1.4 is resistant to calcium-dependent inhibition so channels can stay open longer to support the tonic release of neurotransmitters in the dark (Joiner and Lee, 2015).

Rods and cones use glutamate as the neurotransmitter and signal to both bipolar and horizontal cells, whose processes invaginate the terminal. The benefit of having the synaptic cleft invaginated has been suggested to improve the fidelity of information flow by preventing neurotransmitter spillover (Rao-Mirotznik et al., 1998). The function of the horizontal cell is to aid the integration of PR signaling and through inhibitory feedback, signaling generates the center-surround receptive fields that provide exquisite contrast sensitivity and edge detection to our vision (Thoreson and Mangel, 2012; Boije et al., 2016). The function of the bipolar cells is to relay the light signal through to ganglion cells, the output neuron of the retina. There are 10–13 types of bipolar cells divided into two functional groups, the ON and OFF cells (Masland, 2001). ON-bipolar cells are sign-inverting. They use metabotropic glutamate receptors that signal through a heterotrimeric G-protein cascade to inhibit an ion channel so that they are hyperpolarized in the dark (Martemyanov and Sampath, 2017). When PR hyperpolarize in response to light and glutamate release decreases this inhibition is relieved and the ON-bipolar cell depolarizes. OFF-bipolar cells are sign-conserving. They respond to glutamate in the dark using AMPA or kainite ionotropic glutamate receptors which conduct a depolarizing current (DeVries, 2000; Ichinose and Hellmer, 2016). This complexity of PR synaptic organization allows the relatively simple two receptor (rod vs. cone) system to encode a wide variety of temporal, contrast, and color information. This contrasts with the OSN synapse where the diversity of receptors rather than connections seems to provide detailed information flow. We would be remiss not to point out that the OSN synapse has not been studied at nearly the depth of PR synapses so there could be more similarities than we currently know.

Rod and cone presynaptic terminals are quite distinct (Moser et al., 2020). Rod presynaptic terminals are large, spherical, and contain a single long ribbon. The ribbon curves around the processes invaginating the PR terminal thus creating an extensive active zone. Within each invagination, there are two central dendrites from rod-ON bipolar cells and two flanking processes from horizontal cell axons. The rod ribbon synapse is often mistakenly described as only containing one bipolar dendrite because it requires labor-intensive 3D reconstructions of electron micrographs to visualize the second bipolar dendrite (Tsukamoto and Omi, 2013). Cone presynaptic terminals are long and flat with tens of invaginating ribbon synapses. The ribbons are of variable length but are shorter than the one found in rods. Each invagination is formed by invading horizontal cell dendrites and cone ON-bipolar dendrites. Cone to cone OFF-bipolar cells synapse at flat contact sites, more like conventional synapses, adjacent to the invaginated ribbon synapses.

The defining organelle of a ribbon synapse is the ribbon itself. The molecular core of all ribbons independent of number, shape, or length is a self-organizing structural protein called RIBEYE (Magupalli et al., 2008). The ribbon generally functions to tether hundreds of synaptic vesicles and facilitates their movement to the plasma membrane for fusion. The exact mechanisms employed by ribbons have been a subject of debate and we refer readers to a recent review for a discussion on this topic (Moser et al., 2020). Further complicating the matter, recent studies of RIBEYE knockout animals have reported surprisingly mild or variable defects in synaptic function (Wan et al., 2005; Lv et al., 2016; Maxeiner et al., 2016). However, in these models, the ribbon is not completely absent in all ribbon containing cells, or there remains a “ghost” ribbon to which synaptic vesicles remain tethered. Investigations of the functions and dynamics of ribbons will continue to be a rich area of research for the near future.

Ribbon synapses express many of the same CAZ proteins as conventional synapses (Zanazzi and Matthews, 2009). An additional function for the CAZ complex of ribbon synapses is to anchor the ribbon to the active zone. This is likely accomplished through an array of protein-protein interactions from RIBEYE through bassoon and RIM-BP to Cav1.4 (Dick et al., 2003; Luo et al., 2017; tom Dieck et al., 2005). Earlier we noted that there is a large degree of diversity in the synaptic expression of different CAZ genes or isoforms. One example of a ribbon synapse-specific CAZ protein is Piccolino. Piccolino is a shorter splice isoform of piccolo which is abundant in conventional synapses. Piccolo functions include integrating the active zone complex with actin dynamics and regulating CAZ protein turnover (Ivanova et al., 2016; Torres and Inestrosa, 2018). Piccolino functionally differs from piccolo in that it binds to RIBEYE and participates in forming the extended plate-like structure of the rod ribbon (Regus-Leidig et al., 2014; Müller et al., 2019).

In summary, studies of PR signal transduction are comprehensive and have led to the elucidation of many foundational principles of ciliary-based signal transduction. The state of detailed knowledge concerning the development, structure, and function of PR synapses is still a developing field. As progress is made in this area, we expect to be able to better define the similarities and differences among primary sensory neurons such as PR and OSN.

## Dealing With Damage Through Renewal

OSN and PR are under constant environmental stress, either from inhaled compounds or light damage. To deal with this stress, OSN, and PR both undergo constant renewal. OSN have a limited lifespan averaging only 30 days before undergoing apoptosis (Farbman, 1990; Cowan and Roskams, 2002). These OSN are constantly being replaced by newly developed neurons that arise from the basal cells of the OE. Two groups of stem cells exist in the OE, the quiescent horizontal basal cells which serve as a reserve stem cell population that activates following severe OE injury, and the actively cycling globose basal cells which are the primary progenitor for OSN renewal and recovery following minor injury (Schwob et al., 2017). The development of new OSN takes roughly 10 days as marked by both expressions of mature OSN markers and glomeruli innervation (Liberia et al., 2019). One challenge inherent in this process is the need to constantly rewire the OE to the OB. The mechanisms that guide axon targeting are a rich area of ongoing research, and recent work has suggested that OR present at the axon terminal play a role in axon guidance by directly responding to molecules originating from the OB (Zamparo et al., 2019). Importantly, OSN are unique in their capacity for constant neurogenesis throughout life. In the few other neural populations where neurogenesis occurs, it is typically more limited.

The renewal options for PR is species dependent. In amphibians and fish, new-born PR are added at the ciliary margin at the far periphery of the retina as the eye continues to grow even in adulthood (Harris and Perron, 1998). And Muller glia can be reprogrammed to replace PR in response to acute injury (Hamon et al., 2016; Wan and Goldman, 2016; Langhe et al., 2017). Neither is the case in mammals—a damaged photoreceptor is lost forever. However, in all species, the outer segments undergo constant renewal. New membranes packed with OS structural proteins and the proteins of the phototransduction cascade are added at the base of the OS constantly (Young, 1967). The net length of the OS is maintained by the process of disc shedding where packets of discs are engulfed and digested by the RPE every day, typically in the morning (Young and Bok, 1969; LaVail, 1980). In mice and humans, the rate of disc shedding equates to each photoreceptor having an entirely new OS about every 10 days (Young, 1967; Jonnal et al., 2010). This is an efficient system for PR but places a great deal of stress on the RPE cells that need to clear all the “garbage” from the photoreceptor OS, in addition to the other critical roles they play in keeping PR functioning.

These renewal systems are far from perfect solutions towards a form of eternal cellular life. The very existence of these unique renewal processes, as imperfect as they may be, highlights the importance of keeping OSN and PR functioning in the face of probable environmental damage. That OSN and PR have evolved different renewal mechanisms just emphasizes the parallel nature of these two primary sensory neurons.

## Summary

In this overview of how OSN and PR function we have highlighted the parallels in the anatomy, signaling cascades, modulation of the electrical responses, and organization of the synapse. Yet, there are many, sometimes opposite, aspects of these features. This demonstrates the facility with which evolution has crafted these two different sensory systems. Were we to expand this comparative analysis to other primary sensory neurons such as auditory and vestibular hair cells, gustatory cells, and mechanosensitive neurons, we would likewise find many similarities among the adaptations that serve each sensory modality.

We have done our best to provide a balanced set of information, however, it is likely apparent that far more details are known about PR than about OSN. Prioritizing research on the visual system has been driven by the relative differences in the negative impact on daily life imposed by blindness vs. anosmia. We are proponents of the maxim that specific exceptions help to prove general rules and thus are advocates for continued investigations that will let us make more accurate comparisons among the sensory systems.

Future research directions will hopefully include delving into solving some of the unknowns we highlighted, such as the identity of ion channels that propagate and modulate action potentials in OSN, the molecular makeup of OSN synapses, especially if OSN synapses are less uniform than we currently assume. Even in PR, there are a lot of unknowns regarding how the differing structure and molecular makeup of rod vs. cone synapses support function. We elected to focus this review on adult cells, but a thorough review of how the retina and OE develop, coupled with focused investigations into the molecular drivers of the renewal processes we discussed could advance therapeutic options for neuronal damage and degeneration. Finally, the reductionist approach of focusing on just the primary sensory neurons needs to be complemented with experiments that can lead to a more integrated view of how these individual neurons work in concert with their respective support cells, how selective circuits are formed, how various types of sensory stimuli is processed, and how signals might be modulated under differing environmental conditions. Once the palette of options that have evolved to process sensory information can be fully elucidated, we will be in a stronger position to effectively combat disease and perhaps even expand our capabilities—could we learn to use our sensitivity for odorant profiles to navigate the world with the precision of a rat, or could we modify our PR so we can see in the UV or infrared?

## Author Contributions

All authors contributed to the writing of this review article. Figures were generated by SB. All authors contributed to the article and approved the submitted version.

## Conflict of Interest

The authors declare that the research was conducted in the absence of any commercial or financial relationships that could be construed as a potential conflict of interest.

## Abbreviations

OSN: Olfactory sensory neuron
PR: Photoreceptor
OE: olfactory epithelium
OS: outer segment
IS: inner segment
ER: endoplasmic reticulum
RPE: retinal pigmented epithelium
TGFβ: transforming growth factor-beta
GPCR: G-protein coupled receptor
OR: odorant receptor
GDP: guanosine diphosphate
GTP: guanosine triphosphate
AC3: adenylate cyclase 3
cAMP: cyclic adenosine monophosphate
CNG: cyclic nucleotide-gated
Na: sodium
Ca: calcium
TMEM16B: transmembrane protein 16B
Cl: chloride
NKCC1: Na^+^-K^+^-2Cl^−^ cotransporter I
CaM: calmodulin
AMP: adenosine monophosphate
CaMKII: calmodulin-dependent kinase II
RGS: regulator of G-protein signaling
GRK3: GPCR kinase 3
PKA: protein kinase A
OPN1MW: medium wavelength opsin 1
OPN1LW: long-wavelength opsin 1
OPN1SW: short-wavelength opsin 1
PDE6: phosphodiesterase 6
cGMP: cyclic guanosine monophosphate
GMP: guanosine monophosphate
GRK1: GPCR kinase 1
NCKX: Potassium-dependent sodium-calcium exchanger
GC: guanylate cyclase
GCAP: guanylate cyclase-activating protein
Nav: voltage-gated sodium channel
Kv: voltage-gated potassium channel
Cav: voltage-gated calcium channel
*I*_kx_: non-inactivating voltage-sensitive M-like potassium current
*I*_h_: hyperpolarization-activated current
*I*_KCa_: calcium-activated potassium current
HCN1: hyperpolarization-activated cyclic nucleotide-gated 1
BK: large-conductance calcium-activated potassium channels
AMPA: α-amino-3-hydroxy-5-methyl-4-isoxazolepropionic acid
NMDA: N-Methyl-d-aspartic acid
CAZ: cytomatrix at the active zone
CAST: CAZ-associated structural protein
RIM: Regulating synaptic membrane exocytosis protein
RIM-BP: RIM binding protein.

## References

[B1] Ahn H. S. Black J. A. Zhao P. Tyrrell L. Waxman S. G. Dib-Hajj S. D. (2011). Nav1.7 is the predominant sodium channel in rodent olfactory sensory neurons. Mol. Pain 7:32. 10.1186/1744-8069-7-32 21569247 PMC3101130

[B2] Anvarian Z. Mykytyn K. Mukhopadhyay S. Pedersen L. B. Christensen S. T. (2019). Cellular signalling by primary cilia in development, organ function and disease. Nat. Rev. Nephrol. 15, 199–219. 10.1038/s41581-019-0116-9 30733609 PMC6426138

[B3] Araneda R. C. Peterlin Z. Zhang X. Chesler A. Firestein S. (2004). A pharmacological profile of the aldehyde receptor repertoire in rat olfactory epithelium. J. Physiol. 555, 743–756. 10.1113/jphysiol.2003.058040 14724183 PMC1664868

[B4] Arshavsky V. Y. Burns M. E. (2012). Photoreceptor signaling: supporting vision across a wide range of light intensities. J. Biol. Chem. 287, 1620–1626. 10.1074/jbc.R111.305243 22074925 PMC3265842

[B5] Arshavsky V. Y. Burns M. E. (2014). Current understanding of signal amplification in phototransduction. Cell. Logist. 4:e29390. 10.4161/cl.29390 25279249 PMC4160332

[B6] Arshavsky V. Y. Lamb T. D. Pugh E. N. Jr. (2002). G proteins and phototransduction. Annu. Rev. Physiol. 64, 153–187. 10.1146/annurev.physiol.64.082701.102229 11826267

[B7] Arshavsky V. Y. Wensel T. G. (2013). Timing is everything: GTPase regulation in phototransduction. Invest. Ophthalmol. Vis. Sci. 54, 7725–7733. 10.1167/iovs.13-13281 24265205 PMC3837634

[B8] Arshavsky V. Y. (2003). Protein translocation in photoreceptor light adaptation: a common theme in vertebrate and invertebrate vision. Sci. STKE 2003:Pe43. 10.1126/stke.2003.204.pe43 14560045

[B9] Bader C. R. Bertrand D. Schwartz E. A. (1982). Voltage-activated and calcium-activated currents studied in solitary rod inner segments from the salamander retina. J. Physiol. 331, 253–284. 10.1113/jphysiol.1982.sp014372 7153904 PMC1197749

[B10] Bader C. R. Macleish P. R. Schwartz E. A. (1979). A voltage-clamp study of the light response in solitary rods of the tiger salamander. J. Physiol. 296, 1–26. 10.1113/jphysiol.1979.sp012988 529060 PMC1279061

[B11] Bakalyar H. A. Reed R. R. (1990). Identification of a specialized adenylyl cyclase that may mediate odorant detection. Science 250, 1403–1406. 10.1126/science.2255909 2255909

[B12] Baker S. A. Kerov V. (2013). Photoreceptor inner and outer segments. Curr. Top. Membr. 72, 231–265. 10.1016/B978-0-12-417027-8.00007-6 24210432

[B13] Barnes S. Hille B. (1989). Ionic channels of the inner segment of tiger salamander cone photoreceptors. J. Gen. Physiol. 94, 719–743. 10.1085/jgp.94.4.719 2482325 PMC2228964

[B14] Barnes S. (1994). After transduction: response shaping and control of transmission by ion channels of the photoreceptor inner segments. Neuroscience 58, 447–459. 10.1016/0306-4522(94)90072-8 7513385

[B15] Barrow A. J. Wu S. M. (2009). Low-conductance HCN1 ion channels augment the frequency response of rod and cone photoreceptors. J. Neurosci. 29, 5841–5853. 10.1523/JNEUROSCI.5746-08.200919420251 19420251 PMC2695939

[B16] Baylor D. A. Matthews G. Nunn B. J. (1984). Location and function of voltage-sensitive conductances in retinal rods of the salamander, *Ambystoma tigrinum*. J. Physiol. 354, 203–223. 10.1113/jphysiol.1984.sp015372 6481634 PMC1193408

[B17] Bech-Hansen N. T. Naylor M. J. Maybaum T. A. Pearce W. G. Koop B. Fishman G. A.. (1998). Loss-of-function mutations in a calcium-channel α1-subunit gene in Xp11.23 cause incomplete X-linked congenital stationary night blindness. Nat. Genet. 19, 264–267. 10.1038/947 9662400

[B18] Beech D. J. Barnes S. (1989). Characterization of a voltage-gated K^+^ channel that accelerates the rod response to dim light. Neuron 3, 573–581. 10.1016/0896-6273(89)90267-5 2642011 PMC3858083

[B19] Blanks J. C. Adinolfi A. M. Lolley R. N. (1974). Synaptogenesis in the photoreceptor terminal of the mouse retina. J. Comp. Neurol. 156, 81–93. 10.1002/cne.901560107 4836656

[B20] Bocksteins E. (2016). Kv5, Kv6, Kv8 and Kv9 subunits: no simple silent bystanders. J. Gen. Physiol. 147, 105–125. 10.1002/cne.901560107 26755771 PMC4727947

[B21] Boekhoff I. Inglese J. Schleicher S. Koch W. J. Lefkowitz R. J. Breer H. (1994). Olfactory desensitization requires membrane targeting of receptor kinase mediated by beta gamma-subunits of heterotrimeric G proteins. J. Biol. Chem. 269, 37–40. 8276821

[B22] Boije H. Shirazi Fard S. Edqvist P. H. Hallböök F. (2016). Horizontal cells, the odd ones out in the retina, give insights into development and disease. Front. Neuroanat. 10:77. 10.3389/fnana.2016.00077 27486389 PMC4949263

[B23] Bolz F. Kasper S. Bufe B. Zufall F. Pyrski M. (2017). Organization and plasticity of sodium channel expression in the mouse olfactory and vomeronasal epithelia. Front. Neuroanat. 11:28. 10.3389/fnana.2017.00028 28420967 PMC5376585

[B24] Borisy F. F. Ronnett G. V. Cunningham A. M. Juilfs D. Beavo J. Snyder S. H. (1992). Calcium/calmodulin-activated phosphodiesterase expressed in olfactory receptor neurons. J. Neurosci. 12, 915–923. 10.1523/JNEUROSCI.12-03-00915.1992 1312138 PMC6576063

[B25] Boulton M. Dayhaw-Barker P. (2001). The role of the retinal pigment epithelium: topographical variation and ageing changes. Eye 15, 384–389. 10.1038/eye.2001.141 11450762

[B26] Bringmann A. Pannicke T. Grosche J. Francke M. Wiedemann P. Skatchkov S. N.. (2006). Müller cells in the healthy and diseased retina. Prog. Retin. Eye Res. 25, 397–424. 10.1016/j.preteyeres.2006.05.003 16839797

[B27] Broekhuyse R. M. Tolhuizen E. F. Janssen A. P. Winkens H. J. (1985). Light induced shift and binding of S-antigen in retinal rods. Curr. Eye Res. 4, 613–618. 10.3109/02713688508999993 2410196

[B28] Buck L. Axel R. (1991). A novel multigene family may encode odorant receptors: a molecular basis for odor recognition. Cell 65, 175–187. 10.1016/0092-8674(91)90418-x 1840504

[B29] Bunt-Milam A. H. Saari J. C. Klock I. B. Garwin G. G. (1985). Zonulae adherentes pore size in the external limiting membrane of the rabbit retina. Invest. Ophthalmol. Vis. Sci. 26, 1377–1380. 4044165

[B30] Burns M. E. Arshavsky V. Y. (2005). Beyond counting photons: trials and trends in vertebrate visual transduction. Neuron 48, 387–401. 10.1016/j.neuron.2005.10.014 16269358

[B31] Burns M. E. Mendez A. Chen J. Baylor D. A. (2002). Dynamics of cyclic GMP synthesis in retinal rods. Neuron 36, 81–91. 10.1016/s0896-6273(02)00911-x 12367508

[B32] Bushdid C. Magnasco M. O. Vosshall L. B. Keller A. (2014). Humans can discriminate more than 1 trillion olfactory stimuli. Science 343, 1370–1372. 10.1126/science.1249168 24653035 PMC4483192

[B33] Calvert P. D. Strissel K. J. Schiesser W. E. Pugh E. N. Jr. Arshavsky V. Y. (2006). Light-driven translocation of signaling proteins in vertebrate photoreceptors. Trends Cell. Biol. 16, 560–568. 10.1016/j.tcb.2006.09.001 16996267

[B34] Canteras N. S. Ribeiro-Barbosa E. R. Goto M. Cipolla-Neto J. Swanson L. W. (2011). The retinohypothalamic tract: comparison of axonal projection patterns from four major targets. Brain Res. Rev. 65, 150–183. 10.1016/j.brainresrev.2010.09.006 20863850

[B35] Carr V. M. Menco B. P. Yankova M. P. Morimoto R. I. Farbman A. I. (2001). Odorants as cell-type specific activators of a heat shock response in the rat olfactory mucosa. J. Comp. Neurol. 432, 425–439. 10.1002/cne.1112 11268007

[B36] Chen C. K. Woodruff M. L. Chen F. S. Chen Y. Cilluffo M. C. Tranchina D. (2012). Modulation of mouse rod response decay by rhodopsin kinase and recoverin. J. Neurosci. 32, 15998–16006. 10.1523/JNEUROSCI.1639-12.2012 23136436 PMC3501282

[B37] Chen T. Y. Yau K. W. (1994). Direct modulation by Ca^2+^-calmodulin of cyclic nucleotide-activated channel of rat olfactory receptor neurons. Nature 368, 545–548. 10.1038/368545a0 7511217

[B38] Choi R. Goldstein B. J. (2018). Olfactory epithelium: cells, clinical disorders and insights from an adult stem cell niche. Laryngoscope Investig. Otolaryngol. 3, 35–42. 10.1002/lio2.135 29492466 PMC5824112

[B39] Cowan C. M. Roskams A. J. (2002). Apoptosis in the mature and developing olfactory neuroepithelium. Microsc. Res. Tech. 58, 204–215. 10.1002/jemt.10150 12203699

[B40] Cuschieri A. Bannister L. H. (1975a). The development of the olfactory mucosa in the mouse: electron microscopy. J. Anat. 119, 471–498. 1141050 PMC1231637

[B41] Cuschieri A. Bannister L. H. (1975b). The development of the olfactory mucosa in the mouse: light microscopy. J. Anat. 119, 277–286. 1133096 PMC1231592

[B42] Cygnar K. D. Zhao H. (2009). Phosphodiesterase *1C* is dispensable for rapid response termination of olfactory sensory neurons. Nat. Neurosci. 12, 454–462. 10.1038/nn.2289 19305400 PMC2712288

[B43] Czirják G. Tóth Z. E. Enyedi P. (2007). Characterization of the heteromeric potassium channel formed by kv2.1 and the retinal subunit kv8.2 in *Xenopus oocytes*. J. Neurophysiol. 98, 1213–1222. 10.1152/jn.00493.2007 17652418

[B44] Dawson T. M. Arriza J. L. Jaworsky D. E. Borisy F. F. Attramadal H. Lefkowitz R. J.. (1993). Beta-adrenergic receptor kinase-2 and beta-arrestin-2 as mediators of odorant-induced desensitization. Science 259, 825–829. 10.1126/science.8381559 8381559

[B45] Deeb S. S. (2005). The molecular basis of variation in human color vision. Clin. Genet. 67, 369–377. 10.1111/j.1399-0004.2004.00343.x 15811001

[B46] Demb J. B. (2008). Functional circuitry of visual adaptation in the retina. J. Physiol. 586, 4377–4384. 10.1113/jphysiol.2008.156638 18617564 PMC2614018

[B47] DeVries S. H. (2000). Bipolar cells use kainate and AMPA receptors to filter visual information into separate channels. Neuron 28, 847–856. 10.1016/s0896-6273(00)00158-6 11163271

[B48] Dick O. tom Dieck S. Altrock W. D. Ammermüller J. Weiler R. Garner C. C.. (2003). The presynaptic active zone protein bassoon is essential for photoreceptor ribbon synapse formation in the retina. Neuron 37, 775–786. 10.1016/s0896-6273(03)00086-2 12628168

[B49] Dionne V. E. (2016). Spontaneously active NaV1.5 sodium channels may underlie odor sensitivity. J. Neurophysiol. 116, 776–783. 10.1152/jn.00114.2016 27193318 PMC4992733

[B50] Doucette R. (1990). Glial influences on axonal growth in the primary olfactory system. Glia 3, 433–449. 10.1002/glia.440030602 2148546

[B51] Dubin A. E. Dionne V. E. (1994). Action potentials and chemosensitive conductances in the dendrites of olfactory neurons suggest new features for odor transduction. J. Gen. Physiol. 103, 181–201. 10.1085/jgp.103.2.181 8189204 PMC2216834

[B52] Erskine L. Herrera E. (2014). Connecting the retina to the brain. ASN Neuro 6:1759091414562107. 10.1177/1759091414562107 25504540 PMC4720220

[B53] Farbman A. I. (1990). Olfactory neurogenesis: genetic or environmental controls? Trends Neurosci. 13, 362–365. 10.1016/0166-2236(90)90017-5 1699323

[B54] Firestein S. (2001). How the olfactory system makes sense of scents. Nature 413, 211–218. 10.1038/35093026 11557990

[B55] Foster R. G. Hankins M. W. (2002). Non-rod, non-cone photoreception in the vertebrates. Prog. Retin. Eye Res. 21, 507–527. 10.1016/s1350-9462(02)00036-8 12433375

[B56] Frenz C. T. Hansen A. Dupuis N. D. Shultz N. Levinson S. R. Finger T. E.. (2014). NaV1.5 sodium channel window currents contribute to spontaneous firing in olfactory sensory neurons. J. Neurophysiol. 112, 1091–1104. 10.1152/jn.00154.2014 24872539 PMC4122723

[B57] Gayet-Primo J. Yaeger D. B. Khanjian R. A. Puthussery T. (2018). Heteromeric K(V)2/K(V)8.2 channels mediate delayed rectifier potassium currents in primate photoreceptors. J. Neurosci. 38, 3414–3427. 10.1523/JNEUROSCI.2440-17.2018 29483285 PMC5895036

[B58] Getchell M. L. Chen Y. Ding X. Sparks D. L. Getchell T. V. (1993). Immunohistochemical localization of a cytochrome P-450 isozyme in human nasal mucosa: age-related trends. Ann. Otol. Rhinol. Laryngol. 102, 368–374. 10.1177/000348949310200509 8489167

[B59] Godfrey P. A. Malnic B. Buck L. B. (2004). The mouse olfactory receptor gene family. Proc. Natl. Acad. Sci. U S A 101, 2156–2161. 10.1073/pnas.0308051100 14769939 PMC357068

[B60] Govardovskii V. I. Calvert P. D. Arshavsky V. Y. (2000). Photoreceptor light adaptation. Untangling desensitization and sensitization. J. Gen. Physiol. 116, 791–794. 10.1085/jgp.116.6.791 11099348 PMC2231822

[B61] Hamon A. Roger J. E. Yang X. J. Perron M. (2016). Müller glial cell-dependent regeneration of the neural retina: an overview across vertebrate model systems. Dev. Dyn. 245, 727–738. 10.1002/dvdy.24375 26661417 PMC4900950

[B62] Harris J. A. West A. K. Chuah M. I. (2009). Olfactory ensheathing cells: nitric oxide production and innate immunity. Glia 57, 1848–1857. 10.1002/glia.20899 19455713

[B63] Harris W. A. Perron M. (1998). Molecular recapitulation: the growth of the vertebrate retina. Int. J. Dev. Biol. 42, 299–304. 9654012

[B64] Hart N. S. Mountford J. K. Voigt V. Fuller-Carter P. Barth M. Nerbonne J. M.. (2019). The role of the voltage-gated potassium channel proteins Kv8.2 and Kv2.1 in vision and retinal disease: insights from the study of mouse gene knock-out mutations. eNeuro 6:ENEURO.0032-19.2019. 10.1523/ENEURO.0032-19.2019 30820446 PMC6393689

[B65] Hegg C. C. Irwin M. Lucero M. T. (2009). Calcium store-mediated signaling in sustentacular cells of the mouse olfactory epithelium. Glia 57, 634–644. 10.1002/glia.20792 18942758 PMC2657191

[B66] Heydel J. M. Coelho A. Thiebaud N. Legendre A. Le Bon A. M. Faure P.. (2013). Odorant-binding proteins and xenobiotic metabolizing enzymes: implications in olfactory perireceptor events. Anat. Rec. (Hoboken) 296, 1333–1345. 10.1002/ar.22735 23907783

[B67] Higgins M. K. Oprian D. D. Schertler G. F. (2006). Recoverin binds exclusively to an amphipathic peptide at the N terminus of rhodopsin kinase, inhibiting rhodopsin phosphorylation without affecting catalytic activity of the kinase. J. Biol. Chem. 281, 19426–19432. 10.1074/jbc.M602203200 16675451

[B68] Hubbard R. (1969). Absorption spectrum of rhodopsin: 500 nm absorption band. Nature 221, 432–435. 10.1038/221432a0 5784419

[B69] Hunt D. M. Wilkie S. E. Bowmaker J. K. Poopalasundaram S. (2001). Vision in the ultraviolet. Cell. Mol. Life Sci. 58, 1583–1598. 10.1007/PL00000798 11706986 PMC11337280

[B70] Ichinose T. Hellmer C. B. (2016). Differential signalling and glutamate receptor compositions in the OFF bipolar cell types in the mouse retina. J. Physiol. 594, 883–894. 10.1113/JP271458 26553530 PMC4753269

[B71] Ivanova D. Dirks A. Fejtova A. (2016). Bassoon and piccolo regulate ubiquitination and link presynaptic molecular dynamics with activity-regulated gene expression. J. Physiol. 594, 5441–5448. 10.1113/JP271826 26915533 PMC5043050

[B72] Joiner M. L. Lee A. (2015). Voltage-gated Cav1 channels in disorders of vision and hearing. Curr. Mol. Pharmacol. 8, 143–148. 10.2174/1874467208666150507104937 25966695 PMC4634632

[B73] Jones D. T. Reed R. R. (1989). Golf: an olfactory neuron specific-G protein involved in odorant signal transduction. Science 244, 790–795. 10.1126/science.2499043 2499043

[B74] Jonnal R. S. Besecker J. R. Derby J. C. Kocaoglu O. P. Cense B. Gao W.. (2010). Imaging outer segment renewal in living human cone photoreceptors. Opt. Express. 18, 5257–5270. 10.1364/OE.18.005257 20389538 PMC3113600

[B75] Kawai F. Kurahashi T. Kaneko A. (1996). T-type Ca^2+^ channel lowers the threshold of spike generation in the newt olfactory receptor cell. J. Gen. Physiol. 108, 525–535. 10.1085/jgp.108.6.525 8972390 PMC2229340

[B76] Kawai F. (2002). Ca^2+^-activated K^+^ currents regulate odor adaptation by modulating spike encoding of olfactory receptor cells. Biophys. J. 82, 2005–2015. 10.1016/S0006-3495(02)75549-5 11916858 PMC1301996

[B77] Kern R. C. Pitovski D. Z. (1997). Localization of 11 beta-hydroxysteroid dehydrogenase: specific protector of the mineralocorticoid receptor in mammalian olfactory mucosa. Acta Otolaryngol. 117, 738–743. 10.3109/00016489709113470 9349873

[B78] Kleene S. J. (1993). Origin of the chloride current in olfactory transduction. Neuron 11, 123–132. 10.1016/0896-6273(93)90276-w 8393322

[B79] Knop G. C. Seeliger M. W. Thiel F. Mataruga A. Kaupp U. B. Friedburg C.. (2008). Light responses in the mouse retina are prolonged upon targeted deletion of the HCN1 channel gene. Eur. J. Neurosci. 28, 2221–2230. 10.1111/j.1460-9568.2008.06512.x 19019198

[B80] Komolov K. E. Senin I. I. Kovaleva N. A. Christoph M. P. Churumova V. A. Grigoriev I. I.. (2009). Mechanism of rhodopsin kinase regulation by recoverin. J. Neurochem. 110, 72–79. 10.1111/j.1471-4159.2009.06118.x 19457073

[B81] Kreysing M. Boyde L. Guck J. Chalut K. J. (2010). Physical insight into light scattering by photoreceptor cell nuclei. Opt. Lett. 35, 2639–2641. 10.1364/OL.35.002639 20680084

[B82] Krispel C. M. Chen D. Melling N. Chen Y. J. Martemyanov K. A. Quillinan N.. (2006). RGS expression rate-limits recovery of rod photoresponses. Neuron 51, 409–416. 10.1016/j.neuron.2006.07.010 16908407

[B83] Kurahashi T. Menini A. (1997). Mechanism of odorant adaptation in the olfactory receptor cell. Nature 385, 725–729. 10.1038/385725a0 9034189

[B84] Lamb T. D. (2013). Evolution of phototransduction, vertebrate photoreceptors and retina. Prog. Retin. Eye Res. 36, 52–119. 10.1016/j.preteyeres.2013.06.001 23792002

[B85] Langhe R. Chesneau A. Colozza G. Hidalgo M. Ail D. Locker M.. (2017). Müller glial cell reactivation in *Xenopus* models of retinal degeneration. Glia 65, 1333–1349. 10.1002/glia.23165 28548249

[B86] LaVail M. M. (1980). Circadian nature of rod outer segment disc shedding in the rat. Invest. Ophthalmol. Vis. Sci. 19, 407–411. 7358492

[B87] Lazarevic V. Pothula S. Andres-Alonso M. Fejtova A. (2013). Molecular mechanisms driving homeostatic plasticity of neurotransmitter release. Front. Cell. Neurosci. 7:244. 10.3389/fncel.2013.00244 24348337 PMC3847662

[B88] Leung J. Y. Chapman J. A. Harris J. A. Hale D. Chung R. S. West A. K.. (2008). Olfactory ensheathing cells are attracted to and can endocytose, bacteria. Cell. Mol. Life Sci. 65, 2732–2739. 10.1007/s00018-008-8184-1 18604629 PMC11131851

[B89] Liang F. (2018). Olfactory receptor neuronal dendrites become mostly intra-sustentacularly enwrapped upon maturity. J. Anat. 232, 674–685. 10.1111/joa.12777 29313978 PMC5835782

[B90] Liang F. (2020). Sustentacular cell enwrapment of olfactory receptor neuronal dendrites: an update. Genes (Basel). 11:493. 10.3390/genes11050493 32365880 PMC7291085

[B91] Liberia T. Martin-Lopez E. Meller S. J. Greer C. A. (2019). Sequential maturation of olfactory sensory neurons in the mature olfactory epithelium. eNeuro 6:ENEURO.0266-19.2019. 10.1523/ENEURO.0266-19.2019 31554664 PMC6795559

[B92] Linhares J. M. Pinto P. D. Nascimento S. M. (2008). The number of discernible colors in natural scenes. J. Opt. Soc. Am. A. Opt. Image Sci. Vis. 25, 2918–2924. 10.1364/josaa.25.002918 19037381

[B93] Liu M. Chen T. Y. Ahamed B. Li J. Yau K. W. (1994). Calcium-calmodulin modulation of the olfactory cyclic nucleotide-gated cation channel. Science 266, 1348–1354. 10.1126/science.266.5189.1348 7526466

[B94] London A. Benhar I. Schwartz M. (2013). The retina as a window to the brain-from eye research to CNS disorders. Nat. Rev. Neurol. 9, 44–53. 10.1038/nrneurol.2012.227 23165340

[B95] Lowe G. Gold G. H. (1991). The spatial distributions of odorant sensitivity and odorant-induced currents in salamander olfactory receptor cells. J. Physiol. 442, 147–168. 10.1113/jphysiol.1991.sp018787 1798028 PMC1179883

[B96] Luo F. Liu X. Südhof T. C. Acuna C. (2017). Efficient stimulus-secretion coupling at ribbon synapses requires RIM-binding protein tethering of L-type Ca(2+) channels. Proc. Natl. Acad. Sci. U S A 114, E8081–E8090. 10.1073/pnas.1702991114 28874522 PMC5617259

[B97] Lv C. Stewart W. J. Akanyeti O. Frederick C. Zhu J. Santos-Sacchi J.. (2016). Synaptic ribbons require ribeye for electron density, proper synaptic localization and recruitment of calcium channels. Cell Rep. 15, 2784–2795. 10.1016/j.celrep.2016.05.045 27292637 PMC5334794

[B98] MacLeish P. R. Nurse C. A. (2007). Ion channel compartments in photoreceptors: evidence from salamander rods with intact and ablated terminals. J. Neurophysiol. 98, 86–95. 10.1152/jn.00775.2006 17460105

[B99] Magupalli V. G. Schwarz K. Alpadi K. Natarajan S. Seigel G. M. Schmitz F. (2008). Multiple RIBEYE-RIBEYE interactions create a dynamic scaffold for the formation of synaptic ribbons. J. Neurosci. 28, 7954–7967. 10.1523/JNEUROSCI.1964-08.2008 18685021 PMC6670776

[B100] Majumder A. Pahlberg J. Boyd K. K. Kerov V. Kolandaivelu S. Ramamurthy V.. (2013). Transducin translocation contributes to rod survival and enhances synaptic transmission from rods to rod bipolar cells. Proc. Natl. Acad. Sci. U S A 110, 12468–12473. 10.1073/pnas.1222666110 23836670 PMC3725049

[B101] Malnic B. Godfrey P. A. Buck L. B. (2004). The human olfactory receptor gene family. Proc. Natl. Acad. Sci. U S A 101, 2584–2589. 10.1073/pnas.0307882100 14983052 PMC356993

[B102] Mansergh F. Orton N. C. Vessey J. P. Lalonde M. R. Stell W. K. Tremblay F.. (2005). Mutation of the calcium channel gene Cacna1f disrupts calcium signaling, synaptic transmission and cellular organization in mouse retina. Hum. Mol. Genet. 14, 3035–3046. 10.1093/hmg/ddi336 16155113

[B103] Martemyanov K. A. Sampath A. P. (2017). The transduction cascade in retinal ON-bipolar cells: signal processing and disease. Annu. Rev. Vis. Sci. 3, 25–51. 10.1146/annurev-vision-102016-061338 28715957 PMC5778350

[B104] Mashukova A. Spehr M. Hatt H. Neuhaus E. M. (2006). β-arrestin2-mediated internalization of mammalian odorant receptors. J. Neurosci. 26, 9902–9912. 10.1523/JNEUROSCI.2897-06.2006 17005854 PMC6674477

[B105] Masland R. H. (2001). The fundamental plan of the retina. Nat. Neurosci. 4, 877–886. 10.1038/nn0901-877 11528418

[B106] Matthews G. Fuchs P. (2010). The diverse roles of ribbon synapses in sensory neurotransmission. Nat. Rev. Neurosci. 11, 812–822. 10.1038/nrn2924 21045860 PMC3065184

[B107] Maxeiner S. Luo F. Tan A. Schmitz F. Südhof T. C. (2016). How to make a synaptic ribbon: RIBEYE deletion abolishes ribbons in retinal synapses and disrupts neurotransmitter release. EMBO J. 35, 1098–1114. 10.15252/embj.201592701 26929012 PMC4868958

[B108] McCudden C. R. Hains M. D. Kimple R. J. Siderovski D. P. Willard F. S. (2005). G-protein signaling: back to the future. Cell. Mol. Life Sci. 62, 551–577. 10.1007/s00018-004-4462-3 15747061 PMC2794341

[B109] McIntyre J. C. Williams C. L. Martens J. R. (2013). Smelling the roses and seeing the light: gene therapy for ciliopathies. Trends Biotechnol. 31, 355–363. 10.1016/j.tibtech.2013.03.005 23601268 PMC3665744

[B110] Melien O. (2007). Heterotrimeric G proteins and disease. Methods Mol. Biol. 361, 119–144. 10.1385/1-59745-208-4:119 17172709

[B111] Menco B. P. Birrell G. B. Fuller C. M. Ezeh P. I. Keeton D. A. Benos D. J. (1998). Ultrastructural localization of amiloride-sensitive sodium channels and Na^+^, K^+^-ATPase in the rat’s olfactory epithelial surface. Chem. Senses 23, 137–149. 10.1093/chemse/23.2.137 9589162

[B112] Menco B. P. (1980a). Qualitative and quantitative freeze-fracture studies on olfactory and nasal respiratory epithelial surfaces of frog, ox, rat and dog: III. Tight-junctions. Cell Tissue Res. 211, 361–373. 10.1007/BF00234393 6968243

[B113] Menco B. P. (1980b). Qualitative and quantitative freeze-fracture studies on olfactory and nasal respiratory structures of frog, ox, rat and dog: I. A general survey. Cell Tissue Res. 207, 183–209. 10.1007/BF00234393 6966972

[B114] Mendez A. Burns M. E. Sokal I. Dizhoor A. M. Baehr W. Palczewski K.. (2001). Role of guanylate cyclase-activating proteins (GCAPs) in setting the flash sensitivity of rod photoreceptors. Proc. Natl. Acad. Sci. U S A 98, 9948–9953. 10.1073/pnas.171308998 11493703 PMC55558

[B115] Michalakis S. Becirovic E. Biel M. (2018). Retinal cyclic nucleotide-gated channels: from pathophysiology to therapy. Int. J. Mol. Sci. 19:749. 10.3390/ijms19030749 29518895 PMC5877610

[B116] Mirshahi M. Thillaye B. Tarraf M. de Kozak Y. Faure J. P. (1994). Light-induced changes in S-antigen (arrestin) localization in retinal photoreceptors: differences between rods and cones and defective process in RCS rat retinal dystrophy. Eur. J. Cell. Biol. 63, 61–67. 8005106

[B117] Mollon J. D. (1999). Color vision: opsins and options. Proc. Natl. Acad. Sci. U S A 96, 4743–4745. 10.1073/pnas.96.9.4743 10220361 PMC33565

[B118] Mombaerts P. Wang F. Dulac C. Chao S. K. Nemes A. Mendelsohn M.. (1996). Visualizing an olfactory sensory map. Cell 87, 675–686. 10.1016/s0092-8674(00)81387-2 8929536

[B119] Moriondo A. Pelucchi B. Rispoli G. (2001). Calcium-activated potassium current clamps the dark potential of vertebrate rods. Eur. J. Neurosci. 14, 19–26. 10.1046/j.0953-816x.2001.01605.x 11488945

[B120] Moriondo A. Rispoli G. (2010). The contribution of cationic conductances to the potential of rod photoreceptors. Eur. Biophys. J. 39, 889–902. 10.1007/s00249-009-0419-z 19234695

[B121] Morrison E. E. Costanzo R. M. (1990). Morphology of the human olfactory epithelium. J. Comp. Neurol. 297, 1–13. 10.1002/cne.902970102 2376627

[B122] Moser T. Grabner C. P. Schmitz F. (2020). Sensory processing at ribbon synapses in the retina and the cochlea. Physiol. Rev. 100, 103–144. 10.1152/physrev.00026.2018 31373863

[B123] Müller T. M. Gierke K. Joachimsthaler A. Sticht H. Izsvák Z. Hamra F. K.. (2019). A multiple piccolino-RIBEYE interaction supports plate-shaped synaptic ribbons in retinal neurons. J. Neurosci. 39, 2606–2619. 10.1523/JNEUROSCI.2038-18.2019 30696732 PMC6445989

[B124] Mykytyn K. Askwith C. (2017). G-protein-coupled receptor signaling in cilia. Cold Spring Harb. Perspect. Biol. 9:a028183. 10.1101/cshperspect.a028183 28159877 PMC5585845

[B125] Nakashima N. Ishii T. M. Bessho Y. Kageyama R. Ohmori H. (2013). Hyperpolarisation-activated cyclic nucleotide-gated channels regulate the spontaneous firing rate of olfactory receptor neurons and affect glomerular formation in mice. J. Physiol. 591, 1749–1769. 10.1113/jphysiol.2012.247361 23318872 PMC3624849

[B126] Narusuye K. Kawai F. Miyachi E. (2003). Spike encoding of olfactory receptor cells. Neurosci. Res. 46, 407–413. 10.1016/s0168-0102(03)00131-7 12871762

[B127] Nathans J. Thomas D. Hogness D. S. (1986). Molecular genetics of human color vision: the genes encoding blue, green and red pigments. Science 232, 193–202. 10.1126/science.2937147 2937147

[B128] Nomura T. Takahashi S. Ushiki T. (2004). Cytoarchitecture of the normal rat olfactory epithelium: light and scanning electron microscopic studies. Arch. Histol. Cytol. 67, 159–170. 10.1679/aohc.67.159 15468955

[B129] Norwood J. N. Zhang Q. Card D. Craine A. Ryan T. M. Drew P. J. (2019). Anatomical basis and physiological role of cerebrospinal fluid transport through the murine cribriform plate. eLife 8:e44278. 10.7554/eLife.44278 31063132 PMC6524970

[B130] Nunez-Parra A. Cortes-Campos C. Bacigalupo J. Garcia Mde L. Nualart F. Reyes J. G. (2011). Expression and distribution of facilitative glucose (GLUTs) and monocarboxylate/H^+^ (MCTs) transporters in rat olfactory epithelia. Chem. Senses 36, 771–780. 10.1093/chemse/bjr052 21677031

[B131] Okada M. Erickson A. Hendrickson A. (1994). Light and electron microscopic analysis of synaptic development in Macaca monkey retina as detected by immunocytochemical labeling for the synaptic vesicle protein, SV2. J. Comp. Neurol. 339, 535–558. 10.1002/cne.903390406 8144745

[B132] Omri S. Omri B. Savoldelli M. Jonet L. Thillaye-Goldenberg B. Thuret G.. (2010). The outer limiting membrane (OLM) revisited: clinical implications. Clin. Ophthalmol. 4, 183–195. 10.2147/opth.s5901 20463783 PMC2861922

[B133] Pellegrino R. Sinding C. de Wijk R. A. Hummel T. (2017). Habituation and adaptation to odors in humans. Physiol. Behav. 177, 13–19. 10.1016/j.physbeh.2017.04.006 28408237

[B134] Pelucchi B. Grimaldi A. Moriondo A. (2008). Vertebrate rod photoreceptors express both BK and IK calcium-activated potassium channels, but only BK channels are involved in receptor potential regulation. J. Neurosci. Res. 86, 194–201. 10.1002/jnr.21467 17722068

[B135] Peppel K. Boekhoff I. McDonald P. Breer H. Caron M. G. Lefkowitz R. J. (1997). G protein-coupled receptor kinase 3 (GRK3) gene disruption leads to loss of odorant receptor desensitization. J. Biol. Chem. 272, 25425–25428. 10.1074/jbc.272.41.25425 9325250

[B136] Pilpel Y. Lancet D. (1999). The variable and conserved interfaces of modeled olfactory receptor proteins. Protein Sci. 8, 969–977. 10.1110/ps.8.5.969 10338007 PMC2144322

[B137] Pinto L. H. Klumpp D. J. (1998). Localization of potassium channels in the retina. Prog. Retin. Eye Res. 17, 207–230. 10.1016/s1350-9462(97)00011-6 9695793

[B138] Pridmore R. W. (2014). Orthogonal relations and color constancy in dichromatic colorblindness. PLoS One 9:e107035. 10.1371/journal.pone.0107035 25211128 PMC4161355

[B139] Pugh E. N. Jr. Nikonov S. Lamb T. D. (1999). Molecular mechanisms of vertebrate photoreceptor light adaptation. Curr. Opin. Neurobiol. 9, 410–418. 10.1016/S0959-4388(99)80062-2 10448166

[B140] Pun R. Y. Kleene S. J. (2004). An estimate of the resting membrane resistance of frog olfactory receptor neurones. J. Physiol. 559, 535–542. 10.1113/jphysiol.2004.067611 15272040 PMC1665124

[B141] Pyrski M. Tusty M. Eckstein E. Oboti L. Rodriguez-Gil D. J. Greer C. A.. (2018). P/Q type calcium channel Cav2.1 defines a unique subset of glomeruli in the mouse olfactory bulb. Front. Cell. Neurosci. 12:295. 10.3389/fncel.2018.00295 30233329 PMC6131590

[B142] Ramón-Cueto A. Avila J. (1998). Olfactory ensheathing glia: properties and function. Brain Res. Bull. 46, 175–187. 10.1016/s0361-9230(97)00463-2 9667810

[B143] Rao-Mirotznik R. Buchsbaum G. Sterling P. (1998). Transmitter concentration at a three-dimensional synapse. J. Neurophysiol. 80, 3163–3172. 10.1152/jn.1998.80.6.3163 9862914

[B144] Rebrik T. I. Botchkina I. Arshavsky V. Y. Craft C. M. Korenbrot J. I. (2012). CNG-modulin: a novel Ca-dependent modulator of ligand sensitivity in cone photoreceptor cGMP-gated ion channels. J. Neurosci. 32, 3142–3153. 10.1523/JNEUROSCI.5518-11.2012 22378887 PMC3296131

[B145] Regus-Leidig H. Fuchs M. Löhner M. Leist S. R. Leal-Ortiz S. Chiodo V. A.. (2014). *In vivo* knockdown of Piccolino disrupts presynaptic ribbon morphology in mouse photoreceptor synapses. Front. Cell. Neurosci. 8:259. 10.3389/fncel.2014.00259 25232303 PMC4153300

[B146] Reichenbach A. Bringmann A. (2013). New functions of Müller cells. Glia 61, 651–678. 10.1002/glia.22477 23440929

[B147] Reisert J. Lai J. Yau K. W. Bradley J. (2005). Mechanism of the excitatory Cl- response in mouse olfactory receptor neurons. Neuron 45, 553–561. 10.1016/j.neuron.2005.01.012 15721241 PMC2877386

[B148] Reisert J. Matthews H. R. (1999). Adaptation of the odour-induced response in frog olfactory receptor cells. J. Physiol. 519, Pt 3 801–813. 10.1111/j.1469-7793.1999.0801n.x 10457092 PMC2269541

[B149] Reisert J. (2010). Origin of basal activity in mammalian olfactory receptor neurons. J. Gen. Physiol. 136, 529–540. 10.1085/jgp.201010528 20974772 PMC2964517

[B150] Ressler K. J. Sullivan S. L. Buck L. B. (1993). A zonal organization of odorant receptor gene expression in the olfactory epithelium. Cell 73, 597–609. 10.1016/0092-8674(93)90145-g 7683976

[B151] Rodieck R. W. (1998). The First Steps in Seeing. Sunderland, MA: Sinauer Associates. 562 p.

[B152] Rosenbaum D. M. Rasmussen S. G. Kobilka B. K. (2009). The structure and function of G-protein-coupled receptors. Nature 459, 356–363. 10.1038/nature08144 19458711 PMC3967846

[B153] Rospars J. P. Lánský P. Duchamp A. Duchamp-Viret P. (2003). Relation between stimulus and response in frog olfactory receptor neurons *in vivo*. Eur. J. Neurosci. 18, 1135–1154. 10.1046/j.1460-9568.2003.02766.x 12956713

[B154] Roth L. S. Balkenius A. Kelber A. (2007). Colour perception in a dichromat. J. Exp. Biol. 210, 2795–2800. 10.1242/jeb.007377 17690226

[B155] Saari J. C. (2016). Vitamin A and vision. Subcell. Biochem. 81, 231–259. 10.1007/978-94-024-0945-1_9 27830507

[B156] Sarkar M. A. (1992). Drug metabolism in the nasal mucosa. Pharm. Res. 9, 1–9. 10.1023/a:1018911206646 1589391

[B157] Satir P. Pedersen L. B. Christensen S. T. (2010). The primary cilium at a glance. J. Cell. Sci. 123, 499–503. 10.1242/jcs.050377 20144997 PMC2818190

[B158] Schwob J. E. Jang W. Holbrook E. H. Lin B. Herrick D. B. Peterson J. N.. (2017). Stem and progenitor cells of the mammalian olfactory epithelium: taking poietic license. J. Comp. Neurol. 525, 1034–1054. 10.1002/cne.24105 27560601 PMC5805156

[B159] Seeliger M. W. Brombas A. Weiler R. Humphries P. Knop G. Tanimoto N.. (2011). Modulation of rod photoreceptor output by HCN1 channels is essential for regular mesopic cone vision. Nat. Commun. 2:532. 10.1038/ncomms1540 22068599

[B160] Shichida Y. Matsuyama T. (2009). Evolution of opsins and phototransduction. Philos. Trans. R. Soc. Lond. B Biol. Sci. 364, 2881–2895. 10.1098/rstb.2009.0051 19720651 PMC2781858

[B161] Sinnarajah S. Dessauer C. W. Srikumar D. Chen J. Yuen J. Yilma S.. (2001). RGS2 regulates signal transduction in olfactory neurons by attenuating activation of adenylyl cyclase III. Nature 409, 1051–1055. 10.1038/35059104 11234015

[B162] Sokolov M. Lyubarsky A. L. Strissel K. J. Savchenko A. B. Govardovskii V. I. Pugh E. N. Jr.. (2002). Massive light-driven translocation of transducin between the two major compartments of rod cells: a novel mechanism of light adaptation. Neuron 34, 95–106. 10.1016/s0896-6273(02)00636-0 11931744

[B163] Solomon S. G. Lennie P. (2007). The machinery of colour vision. Nat. Rev. Neurosci. 8, 276–286. 10.1038/nrn2094 17375040

[B164] Solovei I. Kreysing M. Lanctôt C. Kösem S. Peichl L. Cremer T.. (2009). Nuclear architecture of rod photoreceptor cells adapts to vision in mammalian evolution. Cell 137, 356–368. 10.1016/j.cell.2009.01.052 19379699

[B165] Steinke A. Meier-Stiegen S. Drenckhahn D. Asan E. (2008). Molecular composition of tight and adherens junctions in the rat olfactory epithelium and fila. Histochem. Cell. Biol. 130, 339–361. 10.1007/s00418-008-0441-8 18523797

[B166] Stephan A. B. Shum E. Y. Hirsh S. Cygnar K. D. Reisert J. Zhao H. (2009). ANO2 is the cilial calcium-activated chloride channel that may mediate olfactory amplification. Proc. Natl. Acad. Sci. U S A 106, 11776–11781. 10.1073/pnas.0903304106 19561302 PMC2702256

[B167] Strauss O. (2005). The retinal pigment epithelium in visual function. Physiol. Rev. 85, 845–881. 10.1152/physrev.00021.2004 15987797

[B168] Strissel K. J. Lishko P. V. Trieu L. H. Kennedy M. J. Hurley J. B. Arshavsky V. Y. (2005). Recoverin undergoes light-dependent intracellular translocation in rod photoreceptors. J. Biol. Chem. 280, 29250–29255. 10.1074/jbc.M501789200 15961391

[B169] Strotmann J. Breer H. (2011). Internalization of odorant-binding proteins into the mouse olfactory epithelium. Histochem. Cell. Biol. 136, 357–369. 10.1007/s00418-011-0850-y 21818577

[B170] Su C. Y. Menuz K. Carlson J. R. (2009). Olfactory perception: receptors, cells and circuits. Cell 139, 45–59. 10.1016/j.cell.2009.09.015 19804753 PMC2765334

[B171] Su Z. Chen J. Qiu Y. Yuan Y. Zhu F. Zhu Y.. (2013). Olfactory ensheathing cells: the primary innate immunocytes in the olfactory pathway to engulf apoptotic olfactory nerve debris. Glia 61, 490–503. 10.1002/glia.22450 23339073

[B172] Suzuki Y. Takeda M. Farbman A. I. (1996). Supporting cells as phagocytes in the olfactory epithelium after bulbectomy. J. Comp. Neurol. 376, 509–517. 10.1002/(SICI)1096-9861(19961223)376:4<509::AID-CNE1>3.0.CO;2-5 8978466

[B173] Szabadi E. (2018). Functional organization of the sympathetic pathways controlling the pupil: light-inhibited and light-stimulated pathways. Front. Neurol. 9:1069. 10.3389/fneur.2018.01069 30619035 PMC6305320

[B174] Szule J. A. Jung J. H. McMahan U. J. (2015). The structure and function of ‘active zone material’ at synapses. Philos. Trans. R. Soc. Lond. B Biol. Sci. 370:20140189. 10.1098/rstb.2014.0189 26009768 PMC4455758

[B175] Tang J. Tang J. Ling E. A. Wu Y. Liang F. (2009). Juxtanodin in the rat olfactory epithelium: specific expression in sustentacular cells and preferential subcellular positioning at the apical junctional belt. Neuroscience 161, 249–258. 10.1016/j.neuroscience.2009.03.051 19332107

[B176] Tanimoto N. Sothilingam V. Euler T. Ruth P. Seeliger M. W. Schubert T. (2012). BK channels mediate pathway-specific modulation of visual signals in the *in vivo* mouse retina. J. Neurosci. 32, 4861–4866. 10.1523/JNEUROSCI.4654-11.2012 22492042 PMC6620922

[B177] Thoreson W. B. Mangel S. C. (2012). Lateral interactions in the outer retina. Prog. Retin. Eye Res. 31, 407–441. 10.1016/j.preteyeres.2012.04.003 22580106 PMC3401171

[B178] tom Dieck S. Altrock W. D. Kessels M. M. Qualmann B. Regus H. Brauner D.. (2005). Molecular dissection of the photoreceptor ribbon synapse: physical interaction of Bassoon and RIBEYE is essential for the assembly of the ribbon complex. J. Cell. Biol. 168, 825–836. 10.1083/jcb.200408157 15728193 PMC2171818

[B179] Torres V. I. Inestrosa N. C. (2018). Vertebrate presynaptic active zone assembly: a role accomplished by diverse molecular and cellular mechanisms. Mol Neurobiol 55, 4513–4528. 10.1083/jcb.200408157 28685386

[B180] Tsin A. Betts-Obregon B. Grigsby J. (2018). Visual cycle proteins: structure, function and roles in human retinal disease. J. Biol. Chem. 293, 13016–13021. 10.1074/jbc.AW118.003228 30002120 PMC6109927

[B181] Tsukamoto Y. Omi N. (2013). Functional allocation of synaptic contacts in microcircuits from rods *via* rod bipolar to AII amacrine cells in the mouse retina. J. Comp. Neurol. 521, 3541–3555. 10.1002/cne.23370 23749582 PMC4265793

[B182] Vassar R. Ngai J. Axel R. (1993). Spatial segregation of odorant receptor expression in the mammalian olfactory epithelium. Cell 74, 309–318. 10.1016/0092-8674(93)90422-m 8343958

[B183] Villar P. S. Delgado R. Vergara C. Reyes J. G. Bacigalupo J. (2017). Energy requirements of odor transduction in the chemosensory cilia of olfactory sensory neurons rely on oxidative phosphorylation and glycolytic processing of extracellular glucose. J. Neurosci. 37, 5736–5743. 10.1523/JNEUROSCI.2640-16.2017 28500222 PMC6596473

[B184] Wan J. Goldman D. (2016). Retina regeneration in zebrafish. Curr. Opin. Genet. Dev. 40, 41–47. 10.1016/j.gde.2016.05.009 27281280 PMC5135611

[B185] Wan L. Almers W. Chen W. (2005). Two ribeye genes in teleosts: the role of Ribeye in ribbon formation and bipolar cell development. J. Neurosci. 25, 941–949. 10.1523/JNEUROSCI.4657-04.2005 15673675 PMC6725632

[B186] Wang J. O’sullivan M. L. Mukherjee D. Puñal V. M. Farsiu S. Kay J. N. (2017). Anatomy and spatial organization of Müller glia in mouse retina. J. Comp. Neurol. 525, 1759–1777. 10.1002/cne.24153 27997986 PMC5542564

[B187] Wei J. Zhao A. Z. Chan G. C. Baker L. P. Impey S. Beavo J. A.. (1998). Phosphorylation and inhibition of olfactory adenylyl cyclase by CaM kinase II in Neurons: a mechanism for attenuation of olfactory signals. Neuron 21, 495–504. 10.1016/s0896-6273(00)80561-9 9768837

[B188] Weiss J. Pyrski M. Jacobi E. Bufe B. Willnecker V. Schick B.. (2011). Loss-of-function mutations in sodium channel Nav1.7 cause anosmia. Nature 472, 186–190. 10.1038/nature09975 21441906 PMC3674497

[B189] Weiss J. Pyrski M. Weissgerber P. Zufall F. (2014). Altered synaptic transmission at olfactory and vomeronasal nerve terminals in mice lacking N-type calcium channel Cav2.2. Eur. J. Neurosci. 40, 3422–3435. 10.1111/ejn.12713 25195871

[B190] Weitz D. Zoche M. Müller F. Beyermann M. Körschen H. G. Kaupp U. B.. (1998). Calmodulin controls the rod photoreceptor CNG channel through an unconventional binding site in the N-terminus of the beta-subunit. EMBO J 17, 2273–2284. 10.1093/emboj/17.8.2273 9545240 PMC1170571

[B191] Wettschureck N. Offermanns S. (2005). Mammalian G proteins and their cell type specific functions. Physiol. Rev. 85, 1159–1204. 10.1152/physrev.00003.2005 16183910

[B192] Whitlock K. E. (2004). A new model for olfactory placode development. Brain Behav. Evol. 64, 126–140. 10.1159/000079742 15353905

[B193] Williams D. S. Arikawa K. Paallysaho T. (1990). Cytoskeletal components of the adherens junctions between the photoreceptors and the supportive Müller cells. J. Comp. Neurol. 295, 155–164. 10.1002/cne.902950113 2341633

[B194] Xu J. W. Slaughter M. M. (2005). Large-conductance calcium-activated potassium channels facilitate transmitter release in salamander rod synapse. J. Neurosci. 25, 7660–7668. 10.1523/JNEUROSCI.1572-05.2005 16107652 PMC6725409

[B195] Yan C. Zhao A. Z. Bentley J. K. Loughney K. Ferguson K. Beavo J. A. (1995). Molecular cloning and characterization of a calmodulin-dependent phosphodiesterase enriched in olfactory sensory neurons. Proc. Natl. Acad. Sci. U S A 92, 9677–9681. 10.1073/pnas.92.21.9677 7568196 PMC40865

[B196] Yokoyama S. Yang H. Starmer W. T. (2008). Molecular basis of spectral tuning in the red- and green-sensitive (M/LWS) pigments in vertebrates. Genetics 179, 2037–2043. 10.1534/genetics.108.090449 18660543 PMC2516078

[B197] Yoon J. Comerci C. J. Weiss L. E. Milenkovic L. Stearns T. Moerner W. E. (2019). Revealing nanoscale morphology of the primary cilium using super-resolution fluorescence microscopy. Biophys. J. 116, 319–329. 10.1016/j.bpj.2018.11.3136 30598282 PMC6349968

[B198] Young R. W. Bok D. (1969). Participation of the retinal pigment epithelium in the rod outer segment renewal process. J. Cell. Biol. 42, 392–403. 10.1083/jcb.42.2.392 5792328 PMC2107669

[B199] Young R. W. (1967). The renewal of photoreceptor cell outer segments. J. Cell Biol. 33, 61–72. 10.1083/jcb.33.1.61 6033942 PMC2107286

[B200] Yu C. R. Power J. Barnea G. O’Donnell S. Brown H. E. Osborne J.. (2004). Spontaneous neural activity is required for the establishment and maintenance of the olfactory sensory map. Neuron 42, 553–566. 10.1016/s0896-6273(04)00224-7 15157418

[B201] Zamparo I. Francia S. Franchi S. A. Redolfi N. Costanzi E. Kerstens A.. (2019). Axonal odorant receptors mediate axon targeting. Cell Rep. 29, 4334.e7–4348.e7 10.1016/j.celrep.2019.11.099 31875544 PMC6941231

[B202] Zanazzi G. Matthews G. (2009). The molecular architecture of ribbon presynaptic terminals. Mol. Neurobiol. 39, 130–148. 10.1007/s12035-009-8058-z 19253034 PMC2701268

